# Optimized inverse kinematics solutions for a 6-DOF robot

**DOI:** 10.1038/s41598-026-51032-w

**Published:** 2026-05-18

**Authors:** Mustafa Osman Bayoume, Mostafa Abdelgeliel, Elsayed Saber, Hassan El-Gamal

**Affiliations:** 1 Mechanical Engineering Department, College of Engineering, Arab Academy for Science and Technology, Alexandria, Egypt; 2 Electrical and Control Engineering Department, College of Engineering, Arab Academy for Science and Technology, Alexandria, Egypt; 3 Department of Mechanical Engineering, Faculty of Engineering, Alexandria University, Alexandria, Egypt

**Keywords:** Inverse kinematics, Sequential quadratic programming, Branch and bound, Hybrid optimization methods, Robot arm control, Discrete optimization, Continuous optimization, Engineering, Mathematics and computing

## Abstract

Inverse kinematics (IK) for surgical robotic manipulators requires a balance between computational speed, pose accuracy, and robustness to poor initialization and near-singular configurations. This paper presents a controlled comparative evaluation of optimization-based IK solvers for a 6-DOF industrial robot (Viper 650s) under a unified constrained optimization framework with identical robot modelling assumptions, constraints, trajectories, and evaluation metrics. Six methods are investigated, spanning standalone and hybrid techniques: Sequential Quadratic Programming (SQP), Branch and Bound (B&B), Ant Colony Optimization (ACO), and three hybrid configurations (ACO-SQP, B&B-SQP, and a three-stage ACO-B&B-SQP pipeline). All methods are assessed using the same evaluation framework to examine the practical effect of combining global exploration, bounded search-space reduction, and local refinement on path-following accuracy. Two representative trajectories (a planar triangle and a spatial helix) are executed with repeated trials, and performance is evaluated in terms of trajectory execution time and geometric path-tracking error, supplemented by repeated-run statistical summaries. The optimization methods are implemented in MATLAB and tested within the Automation Control Environment (ACE). The results show that the ACO-B&B-SQP pipeline achieved the lowest mean path error on both trajectories under the present setup, whereas SQP remained the fastest. The study is positioned as a unified comparative evaluation framework for optimization-based IK on the Viper 650s robot, incorporating the implementation and evaluation of a three-stage ACO–B&B–SQP hybrid pipeline within this common framework.

## Introduction

Inverse kinematics (IK) is a key problem within the discipline of robotics, involving the computation of the joint motions required to position an end-effector of a robot in a specific pose^1^. Whereas forward kinematics (FK) computes the end-effector position from known joint angles^2^, IK has more complicated properties due to being nonlinear and often under-constrained. Accordingly, several solution methods have been developed and are loosely categorized into three classes, namely analytical methods, numerical methods, and optimization methods.

**Analytical methods** give exact solutions in closed form and are very efficient, which makes them well-suited for tasks that require real-time performance^3^. However, they are typically limited to robots with certain geometries, such as intersecting joint axes, and can struggle in redundant configurations^1,4^.

**Numerical methods**, in contrast, are more adaptable and can handle diverse robot structures^3^. Examples include Jacobian-based techniques like the Jacobian Transpose (JT) and Damped Least Squares (DLS)^1^. Advanced strategies, such as Sequential Quadratic Programming (SQP) combined with the Broyden-Fletcher-Goldfarb-Shanno (BFGS) algorithm, enhance convergence in difficult situations, such as near joint limits or poor initial configurations^5^. Despite their flexibility, numerical methods may require significant computation and can sometimes be unstable^4^.

**Optimization-based approaches** offer a different way to address the problem. Continuous optimization lets parameters vary within specified ranges, using methods such as linear, nonlinear, or quadratic programming, including SQP^6^. In contrast, discrete optimization restricts variables to a limited set, often integers, which makes the problem more complex but is particularly valuable in areas like signal processing and machine learning^7^. Many discrete problems can be simplified through relaxation to continuous forms or specialized algorithms^7^.

***Motivation and gap:***

In surgical manipulation and suturing, IK solvers must balance low latency, high pose accuracy, and robustness to poor initialization and near-singular configurations. These requirements are often conflicting: fast local methods may converge quickly but remain sensitive to initialization, whereas global or discrete search methods are generally more robust but computationally more expensive. Although SQP-based methods, metaheuristic IK solvers, and hybrid global–local approaches have been widely reported in the literature, many prior studies consider only one solver family, employ different robot models, or use non-unified constraints, trajectories, and performance metrics. As a result, direct assessment of runtime-accuracy trade-offs and the practical value of combining global and local search stages remains limited.

Accordingly, the objective of this study is **not** to claim the first global–local hybrid IK approach in general. Instead, the contribution is positioned as a **controlled and unified comparative** evaluation of multiple continuous, discrete, and hybrid IK optimization methods under the same robot model, joint constraints, trajectories, and reporting protocol. Within this common evaluation setting, a three-stage ACO-B&B-SQP pipeline is included to examine the practical effect of combining ACO-based global exploration, B&B-based bounded search refinement, and SQP-based local optimization in a single workflow.

To address this gap, the present study compares SQP, B&B, ACO, ACO-SQP, B&B-SQP, and ACO-B&B-SQP under identical modeling assumptions, constraints, trajectories, and evaluation metrics.

***The main contributions of this work are as follows:***

1. A unified constrained optimization and evaluation framework is developed for the IK of the 6-DOF Viper 650s robot.
2. A hierarchical hybrid three-stage, ACO-B&B-SQP pipeline is implemented and evaluated within this common framework as an explicit hybrid configuration that combines global exploration, bounded search refinement, and local optimization.
3. Six IK methods spanning continuous, discrete, and hybrid optimization strategies are compared under identical robot modeling assumptions, constraints, trajectories, and evaluation criteria.
4. Experiments on two representative trajectories provide a controlled runtime-accuracy comparison across all tested methods and show that the ACO-B&B-SQP pipeline achieved the lowest mean path error under the present setup.

This paper is organized as follows: Section “Introduction” introduces the topic and provides an overview. Section “Literature review” reviews previous work on IK and optimization. Section “Kinematics model” details the kinematic model of the Viper 650s robot. Section “Control algorithms” describes the control algorithm, highlighting the SQP, B&B, and ACO methods, as well as the hybrid approaches, including the proposed ACO-B&B-SQP framework, for solving IK. Section “Results and discussion” presents results and discussion, and Sect. “Conclusion and future work” concludes with the main findings and future research directions.

## Literature review

Over the years, researchers have developed various ways to solve the inverse kinematics (IK) problem in robotic manipulators, and each has advantages and drawbacks.

The **Jacobian Pseudo-inverse (JPI)** was introduced by Whitney in 1969^8^. Essentially, JPI calculates the joint velocities needed to move the end-effector along a desired path using the Moore–Penrose pseudo-inverse of the Jacobian. Its simplicity and tendency to produce solutions with minimal overall joint movement have made it very popular^9,10^,^11^. However, near-singular configurations, JPI may yield abrupt, large joint-velocity changes, which can destabilize the manipulator^12^.

To overcome this limitation, the **Damped Least Squares (DLS)** method was introduced. By adding a damping factor^13,14^, DLS reduces sensitivity near singularities, effectively smoothing the motion and balancing velocity and positional errors^15^. Its performance, however, depends strongly on the choice of damping factor and on the initial joint configuration, which often requires empirical tuning^16^.

For manipulators with more complex or redundant joints, evolutionary algorithms like **Genetic Algorithms (GAs)** provide a different solution. Inspired by natural selection, these algorithms explore a wide range of possible joint configurations^17,18^, which makes them useful when classical methods struggle^19^. Their main drawbacks are slower convergence and limited precision in fine-tuning solutions^20^.

Similarly, **Particle Swarm Optimization (PSO)**—modeled on the flocking behavior of birds^21,22^—can tackle nonlinear, constrained IK problems without requiring gradient information. Yet, it sometimes gets stuck in local optima, which can limit its effectiveness^23^.

**Dynamic Programming (DP)** offers another perspective by breaking the IK problem into overlapping subproblems^24,25^. While this is highly efficient for structured, low-dimensional problems, it quickly becomes impractical for high-dimensional manipulators because of the memory required^24^.

Optimization-based approaches, like **Sequential Quadratic Programming (SQP)**, first developed by Wilson in 1963 and later refined by Han and Powell^26^, are especially effective for smooth, nonlinear problems of small to medium size^27^.

On the other hand, global search methods such as **Branch and Bound (B&B)**, introduced by Land and Doig in 1960^28,29^, are crucial when it comes to finding guaranteed optimal solutions for discrete or combinatorial problems^30^.

**Ant Colony Optimization (ACO)** is a metaheuristic algorithm that was initiated by Marco Dorigo in 1992. It is inspired by the foraging behavior of real ant colonies and classified under swarm intelligence^31,32^. While ants use pheromones, they can indirectly connect themselves with food sources before the next approach toward the nest^33^. For instance, while ants move and deposit pheromones, the shortest paths accumulate pheromones more rapidly due to faster returns and positive feedback, which helps form optimal routes^34,35^.

**B&B-SQP** hybridization is an approach developed for solving complex mixed-integer nonlinear programming problems containing both discrete decision variables and nonlinear constraints. In SQP, sequential quadratic programming permits the solution of relaxed continuous subproblems, whereas B&B regulates the combinatorial impact by partitioning the solution space into subproblems and pruning suboptimal regions through bounds^36,37^.

**ACO-SQP** hybridization remains an under-explored area, as research that directly combines the two types of algorithms is very sparse. Nevertheless, there are indications of this global metaheuristic search^38^. This suggests that combining SQP’s fast local convergence with ACO’s global exploration might work well, and it represents an explicit future research direction for the direct ACO-SQP hybrids.

In addition, a three-stage ACO-B&B-SQP hybrid has received very limited attention in robotic arm inverse kinematics, particularly in controlled comparative studies under identical constraints, cost functions, and trajectories. This motivates its inclusion here as the main hybrid configuration examined in the present comparative framework, designed to combine ACO-based global exploration, B&B-based bounded search refinement, and SQP-based local convergence.

Table 1 positions the present study relative to representative optimization-based IK literature that is closest in spirit to the current work, including SQP-based IK, hybrid global–local IK, and trajectory-oriented constrained optimization approaches. The comparison shows that prior studies have already demonstrated the usefulness of hybrid optimization strategies for IK. However, these works typically focus on a single solver family, a different robot platform, or non-unified experimental settings. Accordingly, the present study is more appropriately framed as a unified comparative evaluation framework that includes the ACO-B&B-SQP three-stage pipeline within the same common setting, rather than as the first instance of hybrid global–local optimization for IK in general.

**Table 1:** Quantitative comparison of representative prior optimization-based inverse-kinematics studies and the present work.

References	Robot / task	Methods	Evaluation setting / constraints	Quantitative results reported in prior work	Statistics / repeated runs	Direct comparability to present work	What this shows relative to the present work	Limitation relative to present work
^39^	UR10 / 6-DOF	**SQP**, **BP-SQP**	Constrained IK; target pose includes position and orientation; target-pose IK rather than trajectory path-following	Closed-form: **100%, 78.8 s**; SQP: **99.5%, 37 s**; BP-SQP: **100%, 4.2 s**	Repeated-run statistical protocol not clearly emphasized in the summarized comparison	**High** for SQP-based constrained IK on a 6-DOF arm	Shows that **SQP-based IK** and **SQP hybrid initialization strategies** already exist; therefore, the contribution of the present paper should not be framed as the first SQP-based IK approach	No unified comparison against **ACO**, **B&B**, and multiple hybrid families; not a trajectory-level repeated-run benchmark
^40^	Redundant planar manipulators / n-DOF	**CLGA** = closed-loop pseudoinverse + GA	Constraint-aware trajectory generation; repeated circular-motion experiments	GA parameters reported explicitly: OLGA population **100/800**, generations **100**; CLGA population **100**, generations **100**; authors reported improved repeatability and positioning for CLGA	Repeated experiments reported, but not with modern inferential statistics in the summarized form	**Medium**	Shows that **global–local hybridization for IK/trajectory generation** is not new in principle	Planar redundant case, not full-pose industrial **6-DOF** IK; no unified runtime/error benchmark like the present work
^41^	5-DOF humanoid robotic arms; simultaneous target reaching	**PSO**, **SSO**, **BHO**	Point-reaching optimization comparison	Worst RMSE: **0.0864** with PSO; longest CT: **7.6521 s** with SSO; best RMSE: **2 × 10**^**–7**^ and shortest CT: **3.0156 s** with BHO	No repeated-run inferential statistical analysis stated in the extracted summary	**Low**	Shows that **quantitative optimization-based IK comparison** already exists in the literature	Different robot, different DOF, and point-reaching rather than full trajectory path-following
^42^	YASKAWA MH5 industrial robot; trajectory/path tracking under joint limits	**BAT**, **GSA**, **PSO**, **WOA**	Path-tracking comparison under joint constraints	BAT average error about **0.03 × 10**^**–4**^ over four trajectories; GSA average error in range **(0.08 to 0.26) × 10^**^**–4**^; Wilcoxon ranking: **BAT 1**, **GSA 2**, **WOA 3**, **PSO 4**	**Yes** — includes **Wilcoxon test** and ranking analysis	**Medium**	Strong evidence that **trajectory-level quantitative comparisons** already exist; novelty should therefore be framed more narrowly	Compares only metaheuristics; does not include **SQP**, **B&B**, or unified continuous/discrete/hybrid benchmarking
^43^	3-DOF redundant robot; triangle, circular, and sine-wave trajectories	**NNGA**, **PSO**	Trajectory tracking in a simpler redundant manipulator	PSO reportedly improved accuracy by **7%** on triangle and **2%** on circular/sine-wave paths; circular trajectory MSE values reported as **2.95343 × 10**^**–5**^ and **1.69283 × 10**^**–5**^	No repeated-run statistical protocol clearly stated in the summarized results	**Low**	Useful prior example of **trajectory-based quantitative comparison**	Much simpler **3-DOF** case; less directly comparable to a full-pose **6-DOF** industrial robot
^44^	Simulated 6-, 8-, and 12-link manipulators; experimental YASKAWA MH5 / 6-link	**e**^**3**^**GSA** hybrid (GSA + PSO-inspired update)	Joint limits included; fitness includes position, orientation, and collision error; single-target and trajectory-following validation	MATLAB on Ryzen 5 / 8 GB; both methods run **20 times**. For 6-link/no-obstacle: mean error improved from **3.42 × 10**^**–6 m**^ to **0.071 × 10**^**–6 m**^, and time fell from **0.339690 s** to **0.060120 s**. Experimental MH5 trajectories used **100 points**	**Yes** — explicitly reports **20 runs**	**High**	Very relevant prior example of a **hybrid IK solver with repeated-run quantitative evaluation**	Different robot and solver family; no **SQP** stage; not a unified comparison across continuous, bounded-search, and metaheuristic methods
^45^	EOD manipulator / 6-DOF; Baxter / 7-DOF	**BODE-CS** hybrid (DE + Cuckoo Search)	Weighted full-pose objective; random-point IK and trajectory tracking	For 6-DOF with **100 random points**: max position error **0.070 mm**, max fitness **0.112**, mean calculation time **7.96 s** over **30 repeats**; for 7-DOF Baxter: max position error **0.0062 mm**; tracked trajectories also reported	**Yes** — **30 repeats** reported	**High**	Confirms that **modern hybrid IK methods with quantitative trajectory-related reporting** already exist	Does not compare against **SQP** or **B&B**; different robot models and benchmark conditions
^46^	PUMA-560 / 6-DOF, Baxter / 7-DOF, KUKA iiwa / 7-DOF; real KUKA YouBot / 5-DOF	**DE-H** = Differential Evolution + selective Jacobian pseudoinverse step	Explicit position and orientation handling with joint boundaries; random pose tests and real-robot validation	For KUKA iiwa: mean fitness **1.713 × 10**^**–3**^, std **9.085 × 10**^**–3**^, best **1.024 × 10**^**–16**^, worst **0.06788**; IK estimation reported as < **1 s per point** on Intel i7 / 16 GB PC	**Yes** — reports **mean, std, best, worst**	**High**	This is strong evidence that **hybrid global–local IK** is already established; the present work should therefore claim novelty more carefully	Not a trajectory-level benchmark across multiple solver families; does not include **SQP** or **B&B** in the same framework
**Present work**	Viper 650 s / 6-DOF; **2D triangle** and **3D helix** path tracking	**SQP, B&B, ACO, ACO-SQP, B&B-SQP, ACO-B&B-SQP**	Unified constrained formulation; explicit quaternion-based orientation error; identical robot model, constraints, trajectories, and metrics across all methods	**20 repeated runs per method**; runtime reported per full trajectory. Proposed **ACO-B&B-SQP** achieved mean path error **1.84441 mm** (triangle) and **1.87155 mm** (helix); **SQP** was fastest at **226.0294 s** (triangle) and **314.969 s** (helix)	Yes — includes repeated runs, run-to-run standard deviation, and approximate inferential estimates (confidence intervals, Welch’s t-test, and Hedges’ g)	**Internal reference**	The main contribution is best framed as a controlled comparative evaluation under identical conditions, together with the implementation and assessment of an explicit three-stage ACO-B&B-SQP pipeline	Contribution should be positioned as **comparative and integrative**, not as a fundamentally new IK theory

A closer look at how SQP, B&B, ACO, B&B-SQP, ACO-SQP, and ACO-B&B-SQP work will be provided in the Control Algorithms section.

## Kinematics model

*A. Mechanical structure*

**Fig. 1:**
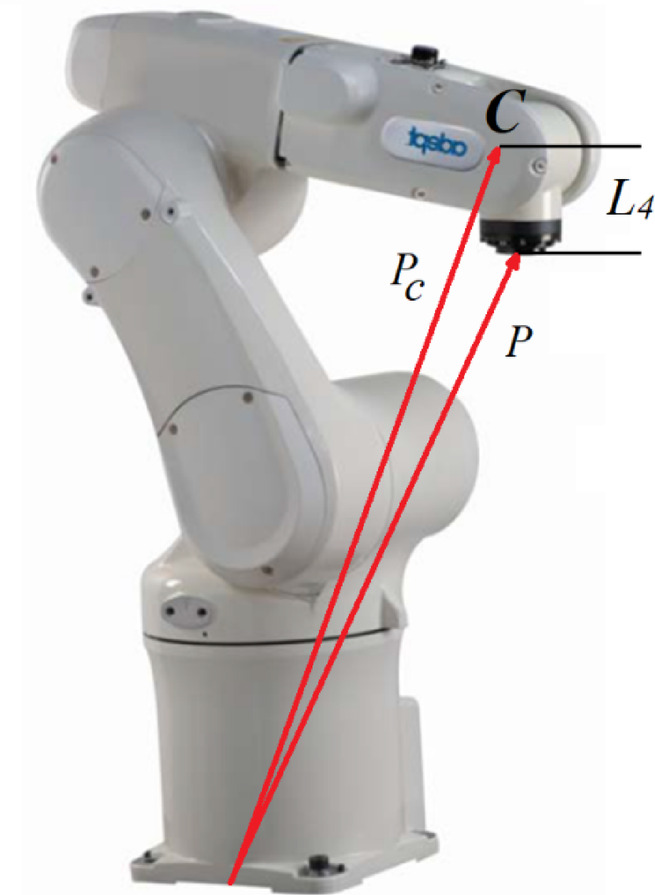
Viper 650s (Point C)^47^.

**Fig. 2:**
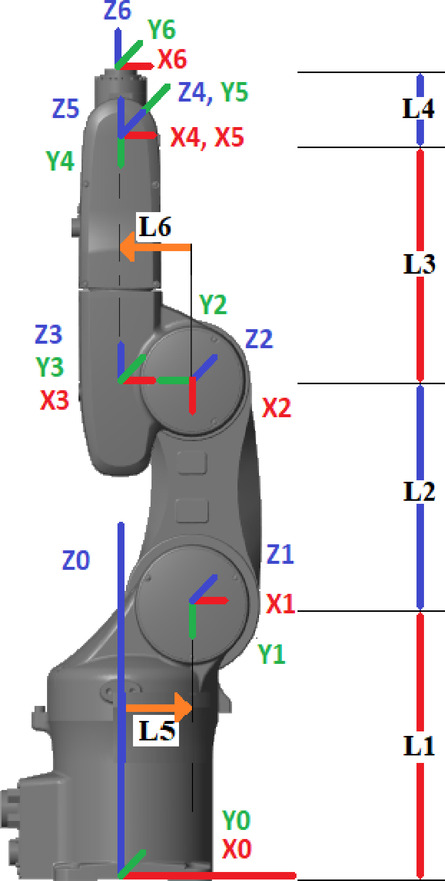
Dimension and assigned coordinate system.

The 6-DOF Viper 650s developed by Adept Co. is shown in Fig. 1, with specifications in Table 2 and its coordinate system in Fig. 2^47^.

**Table 2:** Viper 650s specifications.

Robot dimension (mm)	Joints ranges (Deg)	Joint speeds (Deg/sec)
L1 = 335 L2 = 270 L3 = 295 L4 = 80 L5 = 75 L6 = -90	Joint 1→ ± 170° Joint 2→ −190° to + 45° Joint 3→ −29° to + 265° Joint 4→ ± 190° Joint 5→ ± 120° Joint 6→ ± 360°	Joint 1→ 328 Joint 2→ 300 Joint 3→ 375 Joint 4→ 375 Joint 5→ 375 Joint 6→ 600

Joint limits in Table 2 are reported in degrees for readability; all computations in this work use radians, and the limits are converted to radians before optimization and control.

*B. Forward kinematics (FK)*

The end-effector’s position and orientation are obtained via FK from the robot’s joint values^48^. The matrix method efficiently maps joint space to Cartesian space^49^. The Viper 650s arm applies standard Denavit-Hartenberg parameters, with its coordinate system shown in Fig. 2.

1. *Denavit-hartenberg table of robot arm viper 650s*

The Denavit-Hartenberg (DH) convention, introduced in 1955, is a standard FK method that assigns frames to each link and relates them with transformation matrices^50^. For each joint $n$, frame $n$ is assigned to link $n$ such that ${z}_{n-1}$ is the axis of joint $n$. The relative pose from $n-1$ to $n$ is described by four DH parameters:

- ${\alpha}_{n}$(link twist): the angle from ${z}_{n-1}$ to ${z}_{n}$ measured about ${x}_{n}$.
- ${\theta}_{n}$(joint angle): the angle from ${x}_{n-1}$ to ${x}_{n}$ measured about ${z}_{n-1}$.
- ${a}_{n}$(link length): the distance from ${z}_{n-1}$ to ${z}_{n}$ measured along ${x}_{n}$.
- ${d}_{n}$(link offset): the distance from ${x}_{n-1}$ to ${x}_{n}$ measured along ${z}_{n-1}$.

Table 3 lists the DH parameters of the Viper 650 arm according to the coordinate system in Fig. 2.

**Table 3:** Denavit Hartenberg (DH) table.

Joints	${a}_{n}$(mm)	${\alpha}_{n}$(rad)	${d}_{n}$(mm)	${\theta}_{n}$(rad)
1	L_5_ = 75	−π/2	L_1_ = 335	θ_1_
2	L_2_ = −270	0	0	θ_2_ + π/2
3	L_6_ = −90	+ π/2	0	θ_3−_π/2
4	0	−π/2	L_3_ = 295	θ_4_
5	0	+ π/2	0	θ_5_
6	0	0	L_4_ = 80	θ_6_

2. *End-effector transformation matrix*

The end-effector’s pose is computed using the transformation matrix $T \in {\mathbb{R}}^{4x4}$ for a robot with $n$ joints $n=6$ (3). Its 3 × 3 submatrix R (4) defines orientation, while the last column gives the position vector $\overrightarrow{p}$. From the DH parameters, joint transformations ${}^{0}{T}_{1},\dots ,{}^{5}{T}_{6}$ are derived (1), and their product yields the FK matrix of the end effector, ${}^{0}{T}_{6}$​ (2).

1 $${}^{n-1}{\mathbf{T}}_{n}= \left[\begin{array}{cccc}\mathrm{cos}{\theta}_{n}& -\mathrm{sin}{\theta}_{n}\mathrm{cos}{\alpha}_{n}& \mathrm{sin}{\theta}_{n}\mathrm{sin}{\alpha}_{n}& {a}_{n}\mathrm{cos}{\theta}_{n}\\ \mathrm{sin}{\theta}_{n}& \mathrm{cos}{\theta}_{n}\mathrm{cos}{\alpha}_{n}& -\mathrm{cos}{\theta}_{n}\mathrm{sin}{\alpha}_{n}& {a}_{n}\mathrm{sin}{\theta}_{n}\\ 0& \mathrm{sin}{\alpha}_{n}& \mathrm{cos}{\alpha}_{n}& {d}_{n}\\ 0& 0& 0& 1\end{array}\right]$$

2 $${}^{0}{\mathbf{T}}_{6}={}^{0}{\mathbf{T}}_{1}{}^{1}{\mathbf{T}}_{2}{}^{2}{\mathbf{T}}_{3}{}^{3}{\mathbf{T}}_{4}{}^{4}{\mathbf{T}}_{5}{}^{5}{\mathbf{T}}_{6}$$

3 $${}^{0}{\mathbf{T}}_{6}= {\mathbf{T}}_{n}= \left[\begin{array}{cccc}{n}_{x}& {o}_{x}& {a}_{x}& {p}_{x}\\ {n}_{y}& {o}_{y}& {a}_{y}& {p}_{y}\\ {n}_{z}& {o}_{z}& {a}_{z}& {p}_{z}\\ 0& 0& 0& 1\end{array}\right]$$

4 $$\mathbf{R}= \left[\begin{array}{ccc}{n}_{x}& {o}_{x}& {a}_{x}\\ {n}_{y}& {o}_{y}& {a}_{y}\\ {n}_{z}& {o}_{z}& {a}_{z}\end{array}\right]$$

5 $$\overrightarrow{\mathbf{p}}= \left[\begin{array}{c}{p}_{x}\\ {p}_{y}\\ {p}_{z}\end{array}\right]$$

{n,o,a} are respectively the first, second, and third columns of $\mathbf{R}$ (normal, orientation, approach vectors) used later in the analytical wrist-angle equations.

*C. Inverse kinematics (IK)*

IK calculates joint values for a target end-effector pose^51^. In 6-DOF robots, the analytical decoupling approach assigns the first three joints to positioning and the spherical wrist to orientation^52^.

1. *Position joint angles*

To determine joint angles ${\theta}_{1}$ to ${ \theta }_{3}$, the first link ending at point C (Fig. 1) is considered. The vector $\overrightarrow{{p}_{C}}$ represents the position of C. Given the end-effector target position $\overrightarrow{p}$ in IK, the position of C is derived using Eq. (6).

6 $$\overrightarrow{{\mathbf{p}}_{\mathbf{C}}}= \left[\begin{array}{c}{P}_{Cx}\\ {P}_{Cy}\\ {P}_{Cz}\end{array}\right]=\overrightarrow{\mathbf{p}}-{\mathrm{L}}_{4}*\mathbf{R}*\left[\begin{array}{c}0\\ 0\\ 1\end{array}\right]$$

**Fig. 3:**
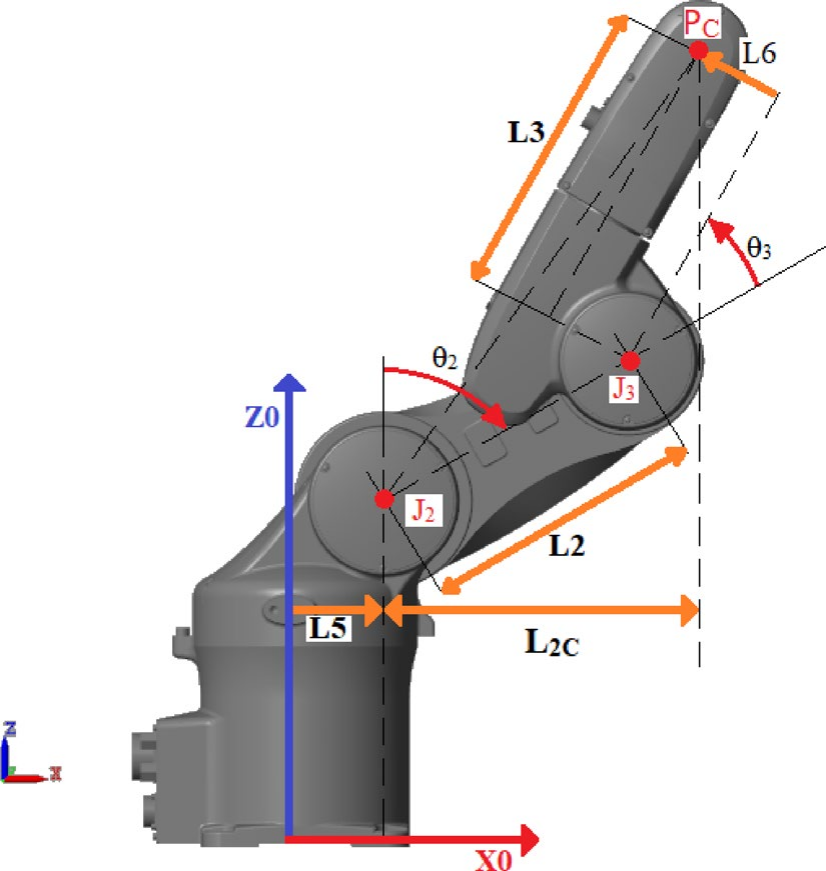
Joint angles from ${{\boldsymbol{\theta}}}_{1}$ To ${{\boldsymbol{\theta}}}_{3}$.

The following equations drive ${\theta}_{1}$ to ${\theta}_{3}$​ for positioning the robot arm using the analytical approach shown in Fig. 3^52^.

7 $${\theta}_{1i} ={\mathrm{tan}}^{-1}\left(\frac{{P}_{Cy}}{{P}_{Cx}}\right)$$

8 $${\theta}_{1ii} = {\theta}_{1i}-\uppi$$

9 $${L}_{2C}= \sqrt{{{P}_{Cx}}^{2}+{{P}_{Cy}}^{2}}- {\mathrm{L}}_{5}$$

10 $${u}_{1}= \sqrt{{L}_{2C}^{2}+{\left({P}_{Cz}-{\mathrm{L}}_{1}\right)}^{2}}$$

11 $${u}_{2}= \sqrt{{\left({L}_{2C}+2*{\mathrm{L}}_{5}\right)}^{2}+{\left({P}_{Cz}-{\mathrm{L}}_{1}\right)}^{2}}$$

12 $$\mathrm{k}= \sqrt{{{\mathrm{L}}_{6}}^{2}+{{\mathrm{L}}_{3}}^{2}}$$

13 $${\theta}_{2i,ii}= \mp \mathrm{a}\mathrm{c}\mathrm{o}\mathrm{s}\left(\frac{{{u}_{1}}^{2}+{{L}_{2}}^{2}-{k}^{2}}{2*{u}_{1}*{L}_{2}}\right)+\mathrm{a}\mathrm{t}\mathrm{a}\mathrm{n}\left(\frac{{L}_{2C}}{{P}_{Cz}-{L}_{1}}\right)$$

14 $${\theta}_{2iii,iv}= \mp \mathrm{a}\mathrm{c}\mathrm{o}\mathrm{s}\left(\frac{{{u}_{2}}^{2}+{{L}_{2}}^{2}-{k}^{2}}{2*{u}_{2}*{L}_{2}}\right)-\mathrm{a}\mathrm{t}\mathrm{a}\mathrm{n}\left(\frac{{L}_{2C}+2*{\mathrm{L}}_{5}}{{P}_{Cz}-{\mathrm{L}}_{1}}\right)$$

15 $${\theta}_{3i,ii}= \pm \mathrm{a}\mathrm{c}\mathrm{o}\mathrm{s}\left(\frac{{{u}_{1}}^{2}-{{\mathrm{L}}_{2}}^{2}-{\mathrm{k}}^{2}}{2*\mathrm{k}*{\mathrm{L}}_{2}}\right)-\mathrm{a}\mathrm{t}\mathrm{a}\mathrm{n}\left(\frac{{\mathrm{L}}_{6}}{{\mathrm{L}}_{3}}\right)$$

16 $${\theta}_{3iii, iv}= \pm \mathrm{a}\mathrm{c}\mathrm{o}\mathrm{s}\left(\frac{{{u}_{2}}^{2}-{{\mathrm{L}}_{2}}^{2}-{\mathrm{k}}^{2}}{2*\mathrm{k}*{\mathrm{L}}_{2}}\right)-\mathrm{a}\mathrm{t}\mathrm{a}\mathrm{n}\left(\frac{{\mathrm{L}}_{6}}{{\mathrm{L}}_{3}}\right)$$

2. *Orientation joint angles*

**Fig. 4:**
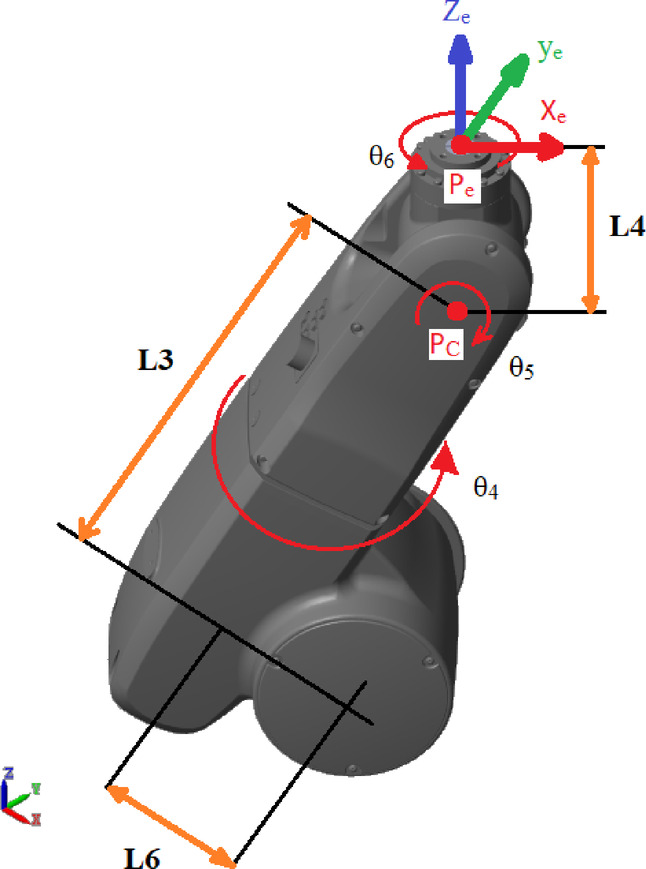
Orientation joint angles.

The equations below determine joints ${\theta}_{4}$ to ${\theta}_{6}$ for controlling the robot orientation, as shown in Fig. 4^52^.

17 $${\theta}_{4p}=\mathrm{a}\mathrm{t}\mathrm{a}\mathrm{n}\left(\frac{{a}_{y}{c}_{1}-{a}_{x}{s}_{1}}{{a}_{x}{c}_{23}{c}_{1}+{a}_{y}{c}_{23}{s}_{1}-{a}_{z}{s}_{23}}\right)$$

18 $${\theta}_{4q}={\theta}_{4p}-\uppi$$

where, $${s}_{n}is \mathrm{sin}\left({\theta}_{n}\right),{c}_{n}is \mathrm{cos}\left({\theta}_{n}\right), {s}_{nm}is \mathrm{sin}\left({\theta}_{n}+{\theta}_{m}\right) and, {c}_{nm}is \mathrm{cos}\left({\theta}_{n}+ {\theta}_{m}\right)$$

19 $${\theta}_{5p}=\mathrm{a}\mathrm{t}\mathrm{a}\mathrm{n}\left(\frac{\sqrt{1-{\left({a}_{x}{s}_{23}{c}_{1}+{a}_{y}{s}_{23}{s}_{1}+{a}_{z}{c}_{23}\right)}^{2}}}{{a}_{x}{s}_{23}{c}_{1}+{a}_{y}{s}_{23}{s}_{1}+{a}_{z}{c}_{23}}\right)$$

20 $${\theta}_{5q}= - {\theta}_{5p}$$

21 $${\theta}_{6p}=\mathrm{a}\mathrm{t}\mathrm{a}\mathrm{n}\left(\frac{{o}_{x}{s}_{23}{c}_{1}+{o}_{y}{s}_{23}{s}_{1}+{o}_{z}{c}_{23}}{-{n}_{x}{s}_{23}{c}_{1}-{n}_{y}{s}_{23}{s}_{1}-{n}_{z}{c}_{23}}\right)$$

22 $${\theta}_{6q}={\theta}_{6p}-\uppi$$

All analytical IK solutions are shown in Table 4.

**Table 4:** All possible robot pose solutions^52^.

Joint/sol	1	2	3	4	5	6	7	8
${\theta}_{1}$	${\theta}_{1i}$	${\theta}_{1i}$	${\theta}_{1ii}$	${\theta}_{1ii}$	${\theta}_{1i}$	${\theta}_{1i}$	${\theta}_{1ii}$	${\theta}_{1ii}$
${\theta}_{2}$	${\theta}_{2i}$	${\theta}_{2ii}$	${\theta}_{2iii}$	${\theta}_{2iv}$	${\theta}_{2i}$	${\theta}_{2ii}$	${\theta}_{2iii}$	${\theta}_{2iv}$
${\theta}_{3}$	${\theta}_{3i}$	${\theta}_{3ii}$	${\theta}_{3iii}$	${\theta}_{3iv}$	${\theta}_{3i}$	${\theta}_{3ii}$	${\theta}_{3iii}$	${\theta}_{3iv}$
${\theta}_{4}$	${\theta}_{4i}$	${\theta}_{4ii}$	${\theta}_{4iii}$	${\theta}_{4iv}$	${\theta}_{4v}$	${\theta}_{4vi}$	${\theta}_{4vii}$	${\theta}_{4viii}$
${\theta}_{5}$	${\theta}_{5i}$	${\theta}_{5ii}$	${\theta}_{5iii}$	${\theta}_{5iv}$	${\theta}_{5v}$	${\theta}_{5vi}$	${\theta}_{5vii}$	${\theta}_{5viii}$
${\theta}_{6}$	${\theta}_{6i}$	${\theta}_{6ii}$	${\theta}_{6iii}$	${\theta}_{6iv}$	${\theta}_{6v}$	${\theta}_{6vi}$	${\theta}_{6vii}$	${\theta}_{6viii}$

## Control algorithms

*A. Steps of control algorithm*

**Fig. 5:**
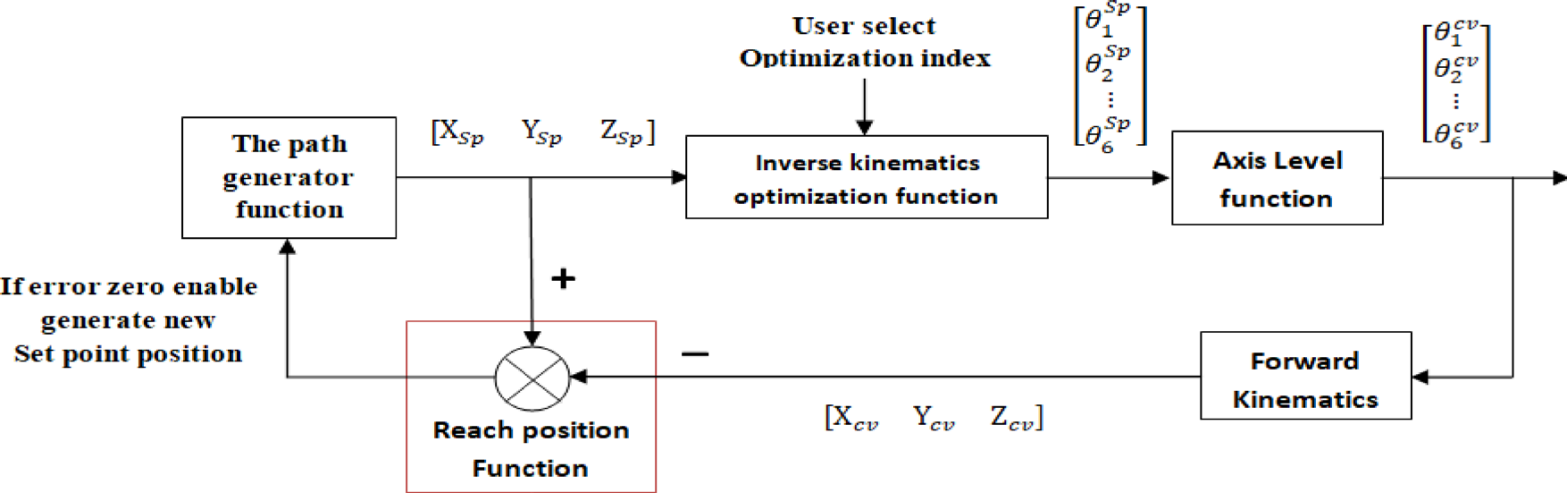
Control block.

The robotic system’s control algorithm comprises multiple levels of operation to render precise motion, as shown in Fig. 5. Starting with the **initialize function**, which sets robot specifications and forms the basis under which control will apply. The **path generation function** sets points of the path and combines with the **inverse kinematics optimization function**, which computes joint angles from set points using SQP or B&B or ACO or hybrid ACO-SQP or hybrid B&B-SQP or hybrid ACO-B&B-SQP, each balancing optimality and computational efficiency. The **axis-level function** uses a common PID-based joint controller and DC motor model to generate joint movements; this control layer is kept fixed across all compared IK methods. The **forward kinematics function** knows the position of the current end effector, while the check the **reach position function** ensures that the deviations stay within limits and signals the path generator to continue. This layered scheme affords high tracking accuracy and a stable operation in proximity to singularities but relies on accurate modeling and can, at times, be computationally intensive.

*B. Control architecture and execution protocol*

To avoid confounding the comparison between inverse-kinematics solvers with differences in low-level robot control, all experiments were executed under the same robot-control architecture and the same ACE execution settings. The Omron Adept Viper s650 robot operated through the eMB-60R controller using the same ACE/eMB-60R execution setup for all runs, while MATLAB was used only to compute the optimized joint references for each trajectory. Therefore, the compared methods differed only in the IK optimization stage; the downstream execution environment remained fixed throughout the study.

At the axis-control level, the same PID-based joint controller and the same DC motor model were used for all experiments. These controller parameters were tuned in preliminary trials to obtain stable motion and were then kept fixed for all six optimization methods and for both trajectories. The fixed PID gains are listed in Table 5. In addition, the same approximately **1 ms** controller execution cycle was maintained throughout all runs, corresponding to an approximately **1 kHz** control/update rate. No method-specific retuning of PID gains, servo update settings, or motor-control parameters was performed.

**Table 5:** Fixed PID gains used in the common axis-level control layer for all experiments.

Joint	${k}_{p}$	${k}_{i}$	${k}_{d}$
Joint 1	1.20	0.020	0.060
Joint 2	1.15	0.020	0.055
Joint 3	1.25	0.022	0.060
Joint 4	0.95	0.015	0.045
Joint 5	0.90	0.015	0.040
Joint 6	0.85	0.012	0.035

The gains in Table 5 correspond to the common axis-level PID controller used uniformly in the execution framework. These parameters, together with the controller update cycle, interpolation settings, and logging procedure, were kept fixed for all methods and were not retuned between experiments.

For trajectory execution, the same Cartesian reference path, the same ACE trajectory-execution interface, and the same controller-side interpolation setting were used for all methods. In particular, the reference commands were executed under the same built-in ACE/eMB-60R interpolation setting for consecutive commanded points throughout all experiments. Each trajectory contained the same 51 nominal reference set-points, and no method-specific changes were introduced in the interpolation pipeline, execution mode, or command update procedure.

During execution, the realized robot motion was recorded using the same acquisition pipeline for all methods, with an approximately **1 ms** logging cycle. The resulting sample counts reported later in the manuscript are therefore consistent with an approximately **1 kHz** logging process over the full trajectory duration. Forward kinematics was then applied offline to reconstruct the realized end-effector pose from the logged robot motion and to compute the geometric path error. In the present study, forward kinematics was used only for state evaluation and post-run error computation; no additional FK-based real-time error-compensation loop, online trajectory correction, or adaptive feedback modification was enabled during trajectory execution. Therefore, the comparison reflects differences in IK optimization under a fixed execution architecture rather than differences in low-level feedback compensation.

A schematic summary of the control flow is as follows: Cartesian reference trajectory → IK optimization method (SQP / B&B / ACO / ACO-SQP / B&B-SQP / ACO-B&B-SQP) → joint reference sequence → common axis-level PID/DC motor control layer and ACE/eMB-60R execution → logged robot motion → forward-kinematics-based evaluation and geometric path-error computation.

*C. General data for all optimization methods*

For all compared inverse-kinematics methods, the same joint variables, desired Cartesian pose, objective function, and joint-limit constraints are used. This unified formulation ensures that the comparison is performed under identical optimization targets, while the methods differ only in their search strategy.

The robot joint-angle vector is defined as

23 $${\boldsymbol{q}}=[{\theta}_{1},{\theta}_{2},{\theta}_{3},{\theta}_{4},{\theta}_{5},{\theta}_{6}{]}^{T}\in {\mathbb{R}}^{6}$$

The common reference joint configuration at trajectory step $t$ is defined as

24 $${{\boldsymbol{q}}}_{t}^{ref}=[{\theta}_{1,t}^{ref},{\theta}_{2,t}^{ref},{\theta}_{3,t}^{ref},{\theta}_{4,t}^{ref},{\theta}_{5,t}^{ref},{\theta}_{6,t}^{ref}{]}^{T}$$

where ${{\boldsymbol{q}}}_{t}^{ref}$ denotes the common reference configuration used for continuity regularization at trajectory step $t$. In this work, for all methods, ${{\boldsymbol{q}}}_{t}^{ref}={{\boldsymbol{q}}}_{{\boldsymbol{t}}-1}$, that is, the accepted joint configuration at the previous executed path point. For the first path point, ${{\boldsymbol{q}}}_{1}^{ref}$ is taken as the measured initial robot configuration. This definition is identical for SQP, B&B, ACO, ACO-SQP, B&B-SQP, and ACO-B&B-SQP.

The current joint-angle configuration during robot execution is denoted by

25 $${{\boldsymbol{q}}}_{{\boldsymbol{c}}{\boldsymbol{v}}}=[{\theta}_{1}^{cv},{\theta}_{2}^{cv},{\theta}_{3}^{cv},{\theta}_{4}^{cv},{\theta}_{5}^{cv},{\theta}_{6}^{cv}{]}^{T}$$

The forward-kinematics model is written as

26 $$T\left(q\right)=FK\left(q\right)= \left[\begin{array}{cc}\mathbf{R}\left(q\right)& p\left(q\right)\\ 0& 1\end{array}\right]$$

where $p\left(q\right)\in {\mathbb{R}}^{3}$ is the end-effector position and $\mathbf{R}\left(q\right)\in SO(3)$ is the corresponding rotation matrix.

The desired end-effector pose at trajectory step $t$ is defined as

27 $${T}_{d,t}=\left[\begin{array}{cc}{\mathbf{R}}_{d,t}& {p}_{d,t}\\ 0& 1\end{array}\right]$$

where ${p}_{d,t}$​ and ${\mathbf{R}}_{d,t}$ are the desired end-effector position and orientation obtained directly from the Cartesian reference trajectory. Thus, the desired Cartesian pose is defined independently of any method-specific joint-space initialization or search variable.

To avoid gimbal lock, orientation error is computed using unit quaternions. Let ${\mathrm{q}}_{R}(q)$ and ${\mathrm{q}}_{R,d,t}$ denote the unit quaternions corresponding to $\mathbf{R}\left(q\right)$ and ${\mathbf{R}}_{d,t}$​, respectively. The orientation error is defined as the geodesic rotation angle (radians):

28 $${e}_{R}(q)=2\mathrm{a}\mathrm{r}\mathrm{c}\mathrm{c}\mathrm{o}\mathrm{s}\left(\left|{\mathrm{q}}_{R,d,t}^{T}{\mathrm{q}}_{R}(q)\right|\right)$$

which gives the minimal rotation angle in [0,π] between the current and desired orientations.

Accordingly, the inverse-kinematics problem at trajectory step $t$ is formulated as.

29 $$\underset{q\in {\mathbb{R}}^{6}}{\mathrm{m}\mathrm{i}\mathrm{n}} {J}_{t}\left(q\right)\mathrm{s}.\mathrm{t}.{{\boldsymbol{q}}}_{{\boldsymbol{m}}{\boldsymbol{i}}{\boldsymbol{n}}} \le {\boldsymbol{q}} \le {{\boldsymbol{q}}}_{{\boldsymbol{m}}{\boldsymbol{a}}{\boldsymbol{x}}}$$

with the common objective function

30 $${J}_{t}(q)=\underset{position error}{{w}_{p}\parallel p(q)-{p}_{d,t}{\parallel }^{2}}+\underset{orientation error}{{w}_{R}{e}_{R}(q{)}^{2}}+(q-{{\boldsymbol{q}}}_{t}^{ref}{)}^{T}Q(q-{{\boldsymbol{q}}}_{t}^{ref})$$

where $${w}_{p}\ge 0$$ weights the translational error ($${\mathrm{m}\mathrm{m}}^{2}$$), $${w}_{R}\ge 0$$ weights the orientation error ($${\mathrm{rad}}^{2}$$), and $${\mathrm{Q}}\underline { \succ }$$$$0$$ is the joint-space regularization matrix. The third term is a continuity regularizer that penalizes deviation from the previous accepted joint configuration $${{\boldsymbol{q}}}_{t}^{ref}$$.

Dimensional consistency between mm and radians is handled through ${\mathrm{w}}_{\mathrm{R}}$ (e.g., selecting ${\mathrm{w}}_{\mathrm{R}}={\mathrm{L}}_{\mathrm{c}}^{2}$ with a characteristic length ${\mathrm{L}}_{\mathrm{c}}$ in mm/rad so that rotational error is scaled to a comparable length-squared magnitude).

Joint limits are imposed as

31 $${q}_{min} \le q \le {q}_{max} (\mathrm{J}\mathrm{o}\mathrm{i}\mathrm{n}\mathrm{t} \mathrm{l}\mathrm{i}\mathrm{m}\mathrm{i}\mathrm{t}\mathrm{s} \mathrm{f}\mathrm{r}\mathrm{o}\mathrm{m} \mathrm{T}\mathrm{a}\mathrm{b}\mathrm{l}\mathrm{e} \mathrm{I}\mathrm{I})$$

32 $${\theta}_{i}^{min}\le {\theta}_{i}\le {\theta}_{i}^{max}, i=1,\dots ,6$$

33 $$g\left( q \right) = \left[ {\begin{array}{*{20}c} {q - q_{\max } } \\ {q_{\min } - q } \\ \end{array} } \right] = \left[ {\begin{array}{*{20}c} {\theta_{1} - \theta_{1}^{\max } } \\ \vdots \\ {\theta_{6} - \theta_{6}^{\max } } \\ {\theta_{1}^{\min } - \theta_{1} } \\ \vdots \\ {\theta_{6}^{\min } - \theta_{6} } \\ \end{array} } \right] \le 0$$

where $q_{min}$ and $q_{max}$ are the minimum and maximum joint limits, $\theta_{i}^{min}$ and $\theta_{i}^{max}$ the allowed range for joint $i$, and $g\left( q \right)$ the inequality constraint function for joint limits.

To avoid ambiguity, the reference configuration $q_{t}^{ref}$ is not method dependent. It is always defined as the previously accepted joint configuration for the current trajectory point. In contrast, quantities such as the SQP initial guess, the ACO discretized candidate set, and the B&B region center are internal search variables only and do not alter the objective function itself. Therefore, all six methods are compared under the same objective function, the same Cartesian target sequence, and the same joint-limit constraints; only the search strategy differs.

*D. Sequential quadratic programming (SQP) mode*

**Fig. 6:**
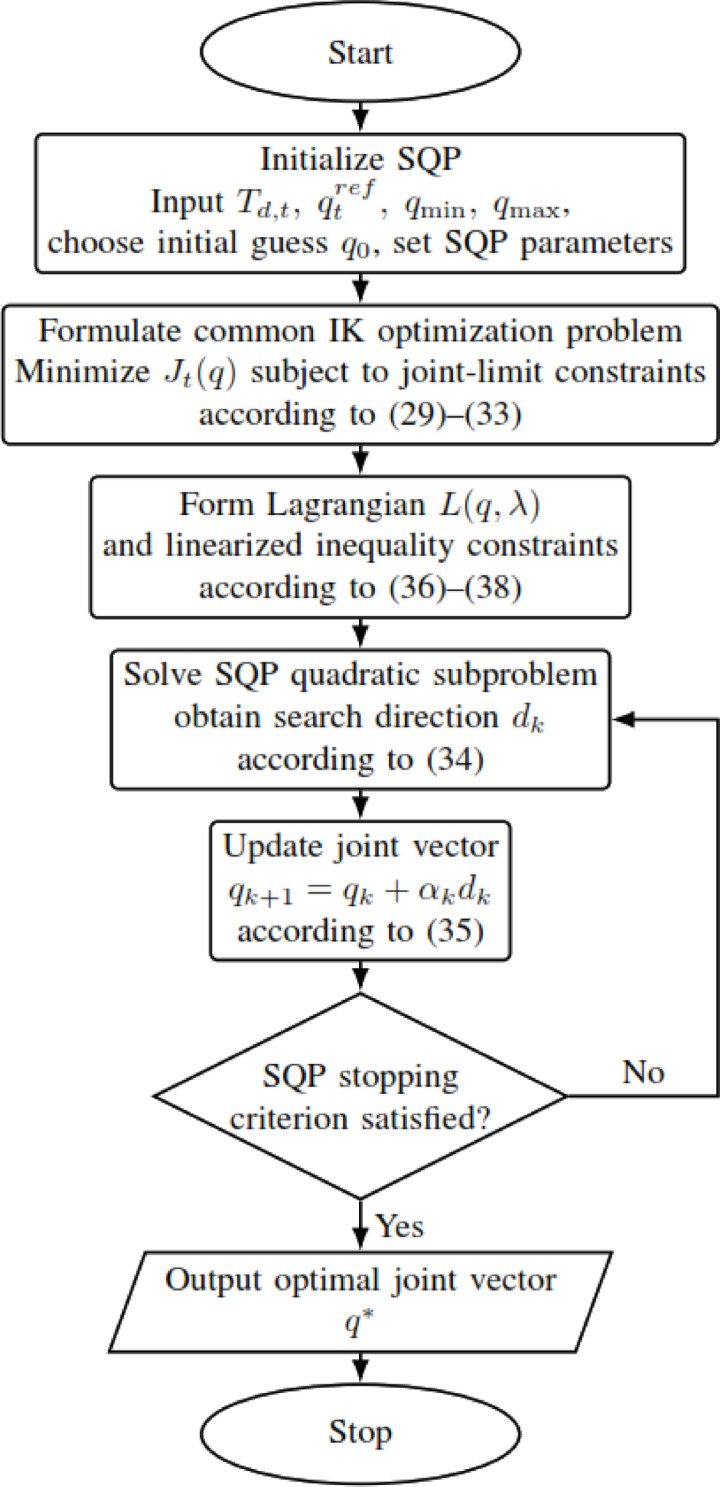
SQP flowchart.

SQP attempts to solve constrained problems through a series of quadratic subproblems, carrying out line searches for global convergence^27,53^; combining Newton steps with Kuhn-Tucker conditions for rapid convergence^54^; and formulating quadratic Lagrangian functions with linear constraints^55^, while using either the actual Hessian or an approximation to establish direction towards the guarantee of convergence^53,55,56^. In the following main mathematical rule with a flowchart shown in Fig. 6:

At iteration $k$, SQP solves:

34 $$\begin{array}{*{20}c} {{\mathrm{min}}} \\ d \\ \end{array} { }\nabla J_{t} \left( {q_{k} } \right)^{{\mathrm{T}}} d + \frac{1}{2}{\mathrm{d}}^{{\mathrm{T}}} { }{\mathbf{H}}_{k} \;d {\mathrm{s}}{\mathrm{.t}}{.}\quad \nabla {\mathbf{g}}_{{\boldsymbol{i}}} \left( {q_{k} } \right)^{{\mathrm{T}}} { }d + {\mathbf{g}}_{{\boldsymbol{i}}} \left( {q_{k} } \right) \le 0$$

where ${\mathrm{H}}_{k}$ is the Hessian matrix or its approximation at iteration $k$, $d$ is the search direction, and $g_{i} \left( q \right)$ denotes the $i$-th inequality constraint. In the present work, the imposed constraints are the joint-limit constraints defined in (31)–(33).

The decision variables are then updated according to

35 $${\boldsymbol{q}}_{k + 1} = {\boldsymbol{q}}_{k} + \alpha_{k} d_{k}$$

where ${\alpha}_{k}$​ is the step size obtained through a line-search procedure, and ${d}_{k}$​ is the solution of the quadratic subproblem at iteration $k$.

The corresponding Lagrangian function is written as

36 $${\mathcal{L}}\left( {q,\lambda } \right) = J_{t} \left( q \right) + \lambda^{T} \left( q \right), \lambda \ge 0$$

with the inequality constraints expressed as

37 $$g_{i} \left( q \right) \le { }0,{ }i = 1, \ldots ,m$$

38 $$\nabla g_{i} \left( {{\mathrm{q}}_{{\mathrm{k}}} } \right)^{{\mathrm{T}}} d + g_{i} \left( {{\mathrm{q}}_{{\mathrm{k}}} } \right) \le 0 {\mathrm{for}} {\mathrm{all}} {\mathrm{i}}$$

For SQP, the initial guess is used only to start the local search; it is not treated as a method-specific desired joint target in the objective. The optimized objective remains the common formulation in (30), with the same continuity reference ${q}_{t}^{ref}$​ used by all other methods.

Because SQP is a local optimization method, it typically converges faster than broader search procedures such as B&B or ACO. However, in non-convex IK problems, its final solution can depend on the starting point and may correspond to a local minimum rather than a globally best solution. This explains why SQP is computationally attractive, but also why it is combined with global-search stages in the hybrid methods considered later.

*E. Branch and Bound (B&B)*

**Fig. 7:**
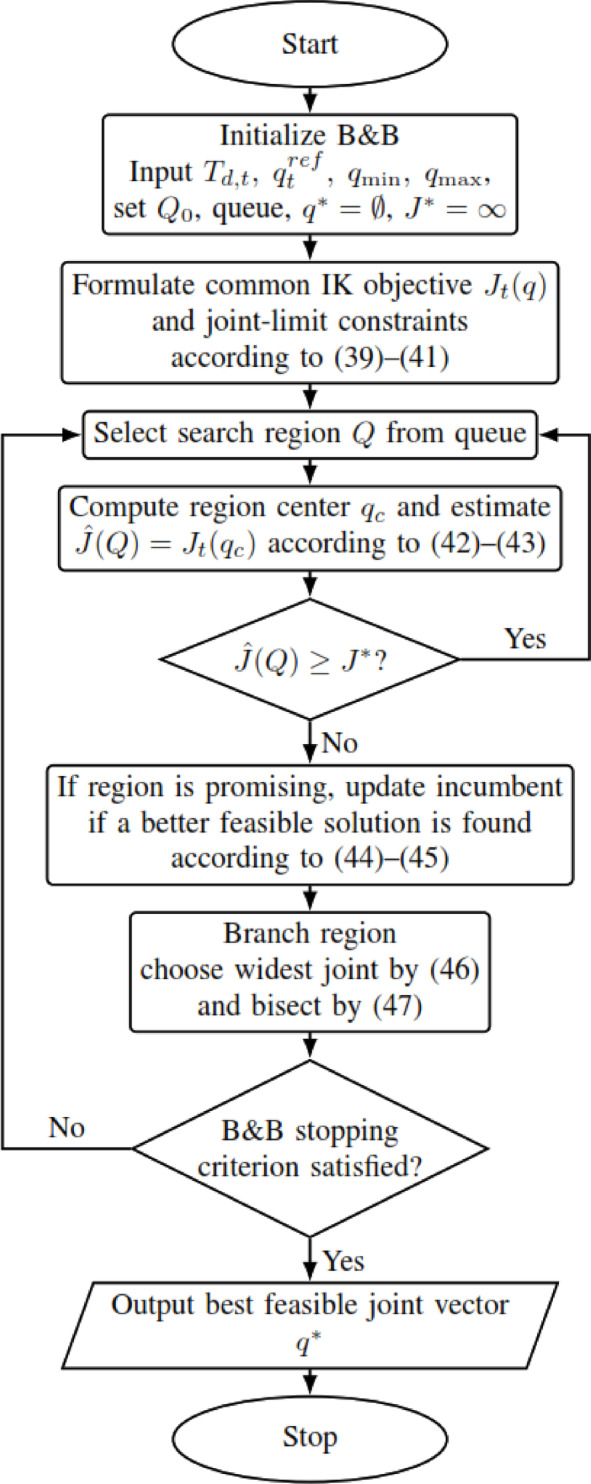
B&B flowchart.

B&B is a core method for discrete and mixed-integer problems (MILPs), exploring the solution space via tree search and pruning^28^. B&B systematically explores the joint space by partitioning it into subregions and heuristically discarding regions that appear unlikely to improve the current best feasible solution. **Branch** divides joint ranges into sub-intervals, while the present implementation evaluates a center-point objective estimate for each region; regions whose estimate is not better than the current incumbent are then heuristically **pruned**^36^. In binary MILPs, fractional variables are split to generate subproblems^57^. Because IK is continuous, the present work employs a B&B-style bounded partition search over continuous joint intervals, combined with local refinement (SQP) to recover a continuous feasible solution. The main mathematical principles with the flowchart shown in Fig. 7 are the following:

For trajectory step $t$, B&B minimizes the same common objective function used by all other methods:

39 $$\underset{q\in {\mathbb{R}}^{6}}{\mathrm{m}\mathrm{i}\mathrm{n} }{J}_{t}(q)$$

40 $${J}_{t}(q)=\underset{position error}{{w}_{p}\parallel p(q)-{p}_{d,t}{\parallel }^{2}}+\underset{orientation error}{{w}_{R}{e}_{R}(q{)}^{2}}+(q-{{\boldsymbol{q}}}_{t}^{ref}{)}^{T}Q(q-{{\boldsymbol{q}}}_{t}^{ref})$$

where $p(q)$ and $R(q)$ are obtained from forward kinematics, ${p}_{d,t}$ and ${R}_{d,t}$​ define the desired end-effector pose at trajectory step $t$, ${e}_{R}(q)$ is the quaternion-based orientation error defined in (28), and ${{\boldsymbol{q}}}_{t}^{ref}$ is the common reference configuration used for continuity regularization. Thus, B&B is evaluated under the same objective function as SQP, ACO, and all hybrid methods; only the search mechanism differs.

The optimization is subject to the same joint-limit constraints defined in (32)–(33). The initial search space is the 6-dimensional hyper-rectangle

41 $${\mathcal{Q}}_{0}=[{\theta}_{1}^{min},{\theta}_{1}^{max}]\times \cdots \times [{\theta}_{6}^{min},{\theta}_{6}^{max}]$$

and the algorithm starts with.

- Best feasible cost: ${J}^{*}=\infty$
- Best feasible solution: ${q}^{*}=\mathrm{\o}$

### Bounding strategy and heuristic pruning

For a sub-region $\mathcal{Q}\subset {\mathbb{R}}^{6}$, let ${\mathrm{q}}_{\mathrm{c}}$ be the center of the region:

42 $${q}_{c}=\frac{{q}_{min}^{\mathcal{Q}}+{q}_{max}^{\mathcal{Q}}}{2}$$

Because the FK-based objective ${J}_{t}(q)$ is nonlinear and non-convex, and no convex relaxation or interval lower-bounding model is derived in the present implementation, the objective value at a single point cannot be treated as a mathematically valid lower bound for the whole region. Therefore, the center-point objective value is used only as a **regional objective estimate**:

43 $$\widehat{J}(\mathcal{Q})={J}_{t}({q}_{c})$$

where $\widehat{J}(\mathcal{Q})$ is interpreted as a heuristic indicator of the quality of region $\mathcal{Q}$, not as a guaranteed lower bound.

The algorithm maintains the most feasible objective value found so far,

44 $${J}^{*}={J}_{t}({q}^{*})$$

where ${q}^{*}$ is the current incumbent feasible solution. Whenever a newly evaluated feasible candidate ${q}_{feas}$​ satisfies ${J}_{t}\left({q}_{feas}\right)<{J}^{*}$, the incumbent solution is updated accordingly.

A region is heuristically pruned whenever

45 $$\widehat{J}\left(\mathcal{Q}\right)\ge {J}^{*}$$

That is, when the objective value at the region center is already not better than the current incumbent. This pruning rule is computationally useful and reproducible, but it does not prove that no better point exists inside $\mathcal{Q}$. Therefore, the present B&B stage should be understood as a heuristic bounded partition search rather than a mathematically exact global branch-and-bound method.

### Branching strategy

At each iteration, the branching variable is selected as the joint with the largest interval width:

46 $${i}^{*}=\mathrm{a}\mathrm{r}\mathrm{g}\underset{i}{\mathrm{m}\mathrm{a}\mathrm{x}}({\theta}_{i}^{max}-{\theta}_{i}^{min})$$

The selected interval is then bisected at its midpoint:

47 $$[{\theta}_{{i}^{*}}^{min},{\theta}_{{i}^{*}}^{max}]\to [{\theta}_{{i}^{*}}^{min},{\theta}_{{i}^{*}}^{mid}]\cup [{\theta}_{{i}^{*}}^{mid},{\theta}_{{i}^{*}}^{max}]$$

which generates two child sub-regions. This choice prioritizes refinement along the least-resolved joint dimension and improves search efficiency.

For B&B, the region center ${q}_{c}$​ is used only for regional evaluation and branching; it is not a method-specific replacement for the common reference configuration ${{\boldsymbol{q}}}_{t}^{ref}$​ in the objective. Accordingly, the present B&B procedure should be interpreted as a heuristic search strategy rather than a certified global optimization algorithm.

### Stopping criteria and computational complexity

The B&B search terminates when one of the following conditions is satisfied:

- the number of explored nodes reaches the maximum limit ${N}_{node,max}={10}^{4}$,
- the maximum interval width in the current region becomes smaller than $\Delta {\theta}_{min}={10}^{-3}$ rad,
- the search queue becomes empty.

These stopping criteria prevent excessive search depth while maintaining a practically useful joint-space resolution.

From a computational perspective, the worst-case complexity of B&B is exponential. If no pruning occurs and each node is split into two child nodes, the number of visited nodes grows with search depth $d$ as

48 $${N}_{nodes}=O({2}^{d})$$

which is a standard property of branch-and-bound-type methods. In practice, however, the actual runtime depends on the number of visited nodes and the cost of evaluating the objective function. If ${N}_{visited}$​ denotes the number of visited nodes and ${C}_{J}$ denotes the cost of one objective-function evaluation, then the practical runtime can be expressed as

49 $${T}_{B\&B}=O({N}_{visited}{C}_{J})$$

where typically ${N}_{visited}\ll {2}^{d}$ when heuristic pruning is effective. This explains why B&B provides broader search coverage than SQP, but at the cost of higher computational effort.

A limitation of the present B&B implementation is that its region-screening criterion is based on a center-point objective estimate rather than a mathematically rigorous lower bound; therefore, the method should be interpreted as a practical heuristic bounded-search strategy rather than a certified global optimization algorithm.

*F. Ant colony optimization (ACO) mode*

**Fig. 8:**
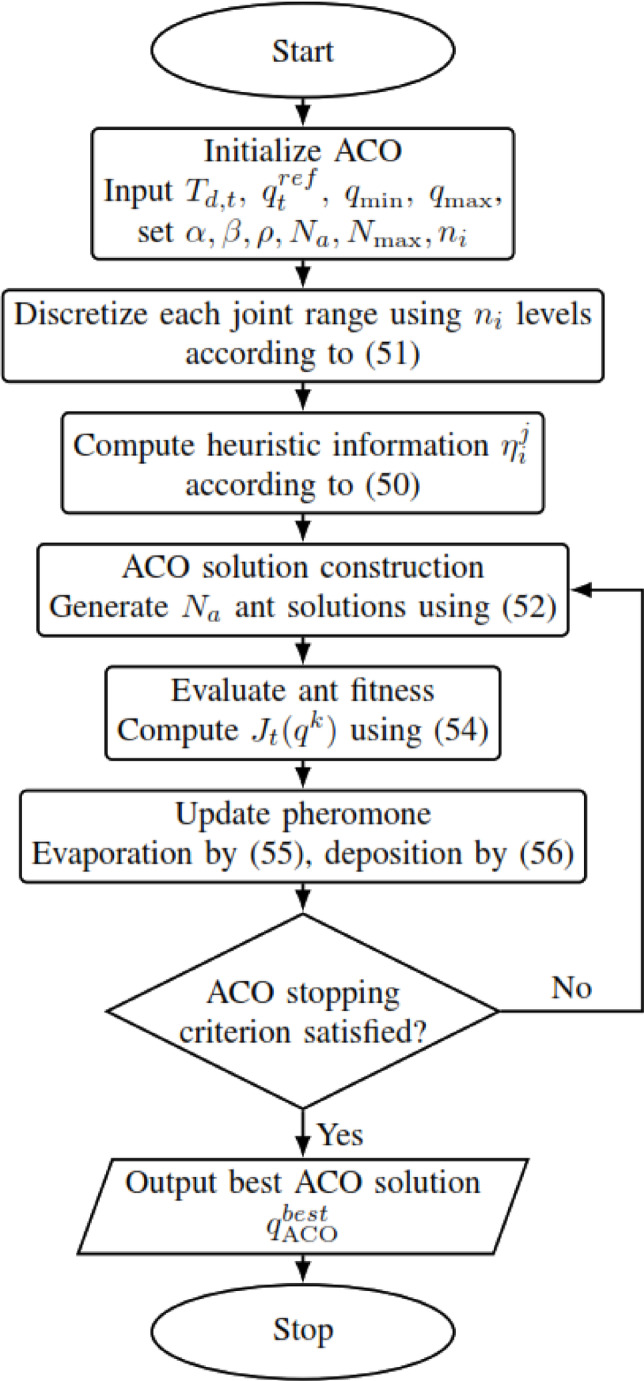
ACO flowchart.

The ACO algorithm is an iterative probabilistic method inspired by the pheromone-guided communication that takes place in the natural world in real ant colonies^58,59^. On the building of solutions, artificial ants build candidate solutions stepwise through a graph, wherein they select components in accordance with transition probabilities that matrix pheromone intensities with heuristic information^60,61^. On completion of the generation of solutions, pheromone levels are updated according to the quality of the solutions, reinforcing good paths while allowing others to lose influence through evaporation^62,63^. The ACO creates equilibrium between exploration and exploitation so that the ACO can converge toward optimal or near-optimal solutions by virtue of collective intelligence^63,64^. The main mathematical principles with the flowchart shown in Fig. 8 are the following:

### Heuristic information

The heuristic information $\eta$ is defined as the inverse of the distance from the common reference configuration:

50 $${\eta}_{i}^{j}=\frac{1}{\left|{\theta}_{i}^{j}- {\theta}_{i,t}^{ref}\right|+\epsilon }$$

where ${\theta}_{i,t}^{ref}$​ is the $i$-th component of the common reference configuration ${{\boldsymbol{q}}}_{t}^{ref}$​, and $\epsilon >0$ is a small constant introduced to avoid division by zero. This heuristic biases the discrete search toward continuity with the previously accepted joint configuration while preserving the same underlying optimization objective across all compared methods.

### Discretization of the search space

Each joint $i$ is defined over the admissible range ${\theta}_{i}\in \left[{\theta}_{i}^{min},{\theta}_{i}^{max}\right]$ which is uniformly discretized into ${n}_{i}$​ levels according to

51 $${\theta}_{i}^{j}={\theta}_{i}^{min}+\left(j-1\right)\frac{{\theta}_{i}^{max}-{\theta}_{i}^{min}}{{n}_{i}-1}, j=1,2,...,{n}_{i}$$

This discretization converts the continuous IK search space into a finite set of candidate joint values suitable for ACO-based exploration.

### Ant solution construction

Each ant constructs a candidate joint vector ${q}^{k}=\left[{\theta}_{1}^{k},\dots ,{\theta}_{6}^{k}\right]$ by selecting one discrete value for each joint according to the transition probability

52 $${P}_{i}^{j}=\frac{{\left({\tau}_{i}^{j}\right)}^{\alpha }{\left({\eta}_{i}^{j}\right)}^{\beta }}{\sum_{l=1}^{{n}_{i}}{\left({\tau}_{i}^{l}\right)}^{\alpha }{\left({\eta}_{i}^{l}\right)}^{\beta }}$$

where ${\tau}_{i}^{j}$​ is the pheromone level associated with the $j$-th discrete value of joint $i$, ${\eta}_{i}^{j}$​ is the corresponding heuristic information, $\alpha$ controls the influence of pheromone, and $\beta$ controls the influence of heuristic information.

Although the IK problem is continuous, ACO requires a discrete candidate set. Therefore, each joint range $\left[{\theta}_{i}^{min},{\theta}_{i}^{max}\right]$ is discretized into ${n}_{i}$​ uniform levels, giving a step size of

53 $$\Delta {\theta}_{i}=\frac{{\theta}_{i}^{max}-{\theta}_{i}^{min}}{{n}_{i}-1}$$

In all experiments, each joint was discretized using ${n}_{i}=51$ uniformly spaced levels. This gives a nominal combinatorial search space of ${51}^{6}\approx 1.76\times {10}^{10}$ discrete joint combinations. The present ACO implementation does not attempt to exhaustively explore that space. Instead, it performs a guided stochastic sampling procedure using ${N}_{a}=30$ ants and ${N}_{max}=100$ iterations, that is, at most 3000 candidate joint vectors before termination, with sampling biased by pheromone reinforcement and the continuity-based heuristic in (50). Accordingly, the ACO stage should be interpreted as a seed-generation mechanism for identifying promising regions of the IK search space rather than as an exhaustive global search over all discretized combinations.

The choice ${n}_{i}=51$ was made as a practical compromise between search resolution and computational cost. A finer discretization increases the chance of generating a seed closer to a good solution basin, but it also increases the candidate-space size and the cost of probabilistic sampling and pheromone updating. A coarser discretization reduces computational burden but may provide poorer seeds for subsequent refinement. In the present framework, the discretization is used only to generate candidate seeds; final solution accuracy is recovered later in continuous space by the SQP stage in the hybrid methods. Therefore, the grid resolution does not need to represent the final IK precision directly; it only needs to be sufficiently dense to identify promising neighborhoods for local refinement.

A practical reason why this moderate discretization can still perform well is that the objective function is not an arbitrary combinatorial landscape. Because it is built from forward-kinematics position error, quaternion orientation error, and a continuity regularization term relative to the previous accepted configuration, neighboring joint configurations often have correlated objective values. Under such conditions, a moderately coarse grid can still locate candidate regions associated with favorable IK branches or smoother local basins, even if it does not sample the exact optimum. The subsequent continuous SQP refinement then improves these seeds to a locally optimized feasible solution. This interpretation is also consistent with the sensitivity study reported later: increasing ${n}_{i}$ beyond the selected value increased runtime, whereas the main qualitative conclusions of the study did not materially change under moderate parameter perturbations.

### ACO termination and practical convergence

Because ACO is a metaheuristic, it does not guarantee global optimality. In this work, practical convergence is assumed when either the maximum number of iterations ${N}_{max}=100$ is reached, or when the best objective improvement falls below ${\upvarepsilon}_{J}={10}^{-6}$ for ${N}_{stall}=15$ consecutive iterations.

### ACO parameter reporting and sensitivity

The ACO parameters $\alpha$, $\beta$, $\rho$ and ${N}_{a}$​ were selected through preliminary tuning on representative target poses in order to balance runtime and final pose error. The final hyperparameter values used in all experiments were $\alpha =1$, $\beta =2$, $\rho =0.1$, ${N}_{a}=30$, ${N}_{max}=100$, ${N}_{stall}=15$, $\upvarepsilon ={10}^{-6}$, ${\upvarepsilon}_{J}={10}^{-6}$, ${Q}_{ph}=1$, and ${n}_{i}=51$. A brief one-factor-at-a-time sensitivity check was also performed by varying $\rho$ by ± 50%, $\beta$ by ± 1, and the number of ants by ± 10. The selected setting provided the best trade-off between runtime and final pose error.

### Evaluate fitness

The fitness of each ant solution is evaluated using the same common objective function adopted by all methods:

54 $${J}_{t}({q}^{k})=\underset{position error}{{w}_{p}\parallel p({q}^{k})-{p}_{d,t}{\parallel }^{2}}+\underset{orientation error}{{w}_{R}{e}_{R}({q}^{k}{)}^{2}}+({q}^{k}-{{\boldsymbol{q}}}_{t}^{ref}{)}^{T}Q({q}^{k}-{{\boldsymbol{q}}}_{t}^{ref})$$

Forward kinematics $FK({q}^{k})$ provides the end-effector position $p({q}^{k})$ and rotation matrix $R({q}^{k})$, and the corresponding unit quaternion ${{\boldsymbol{q}}}_{R}({q}^{k})$ is used to compute the orientation error ${e}_{R}({q}^{k})$ according to (28). Therefore, all ACO candidates are evaluated under the same objective function, the same continuity regularization term, and the same joint-limit constraints used by SQP, B&B, and the hybrid methods.

#### Update pheromone

After evaluating all ant solutions, pheromone values are updated in two stages.

First, pheromone evaporation is applied:

55 $${\tau}_{i}^{j}\leftarrow \left(1-\rho \right){\tau}_{i}^{j}$$

where $\rho$ is the pheromone evaporation rate.

Second, pheromone is deposited along the discrete joint values used by the best-performing ants:

56 $${\tau}_{i}^{j}\leftarrow {\tau}_{i}^{j}+\sum_{k\in best ants}\frac{{Q}_{ph}}{{J}_{t}\left({q}^{k}\right)}$$

where ${Q}_{ph}$​ is a pheromone scaling factor. In this way, discrete joint values associated with lower objective values receive stronger reinforcement and become more likely to be selected in subsequent iterations.

For ACO, the discretized joint candidates are internal search variables only; they are not treated as method-specific desired joint targets in the objective function.

*G. Hybrid ACO–SQP mode*

**Fig. 9:**
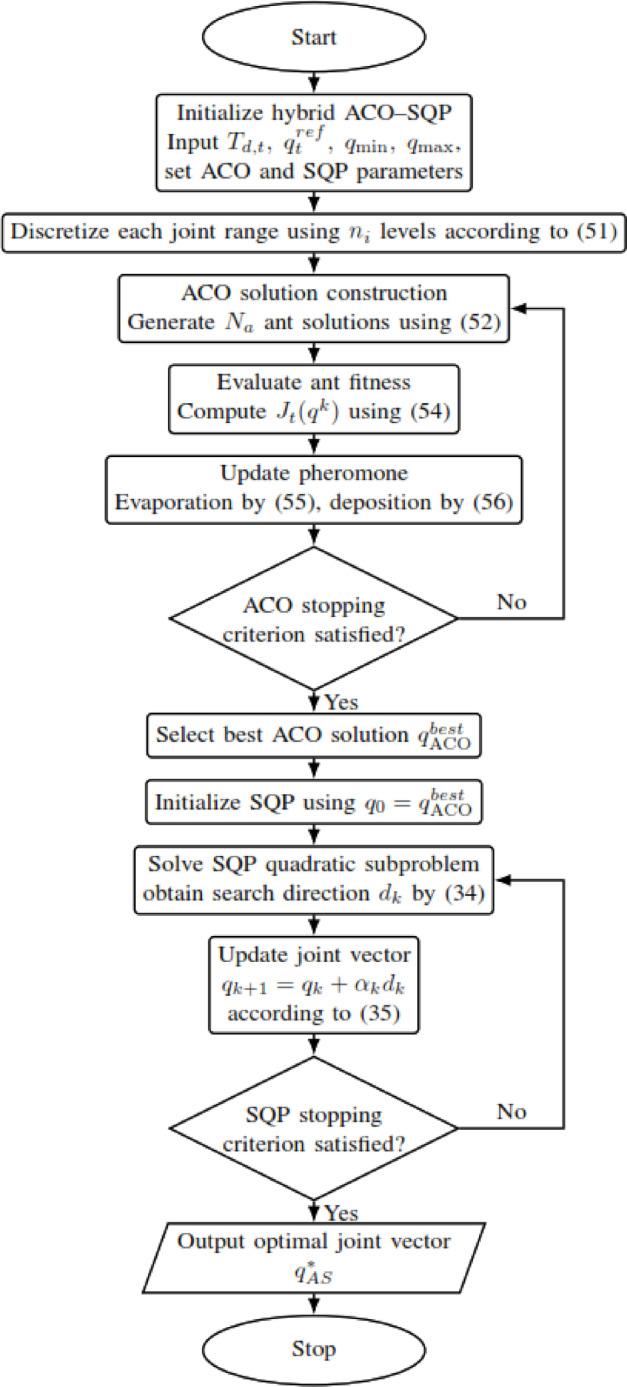
ACO-SQP flowchart.

The proposed hybrid ACO–SQP method is a two-stage global–local optimizer. ACO first performs coarse global exploration on the discretized joint space. SQP then performs continuous local refinement from the best ACO candidate to obtain the final IK solution. For fairness, this hybrid method uses the same desired Cartesian pose ${T}_{d,t}$​, the same continuity reference ${{\boldsymbol{q}}}_{t}^{ref}$, the same joint-limit constraints, and the same objective function ${J}_{t}(q)$ as the other compared methods; only the search procedure differs. The mathematical steps of the proposed method, together with the corresponding flowchart, are shown in Fig. 9.

**Step 1. Define the common inverse-kinematics optimization problem:** Formulate the 6-DOF inverse-kinematics problem using the joint vector $q$ in (23), the common reference configuration ${q}_{t}^{ref}$ in (24), the desired end-effector pose ${T}_{d,t}$ in (27), and the forward-kinematics model in (26). The optimization objective is the common function ${J}_{t}\left(q\right)$ in (30), minimized subject to the joint-limit constraints in (31)–(33).

**Step 2. Run the ACO stage over the full discretized joint space:** Initialize the ACO parameters and discretize each admissible joint interval according to (51). Then construct ant solutions using the continuity-based heuristic in (50), the transition probabilities in (52), the fitness function in (54), and the pheromone-update rules in (55)–(56).

**Step 3. Evaluate the feasible ACO solutions and retain the best candidate:** For each ant solution ${q}^{k}$, evaluate the forward kinematics using (26), compute the quaternion-based orientation error using (28), and evaluate the common objective value using (54). After the ACO stage terminates, sort the feasible ACO solutions and retain the best one as ${q}_{\mathrm{A}\mathrm{C}\mathrm{O}}^{best}$, where ${q}_{\mathrm{A}\mathrm{C}\mathrm{O}}^{best}$ denotes the best feasible solution returned by the ACO stage.

**Step 4. Initialize the SQP stage using the best ACO solution:** Use ${q}_{\mathrm{A}\mathrm{C}\mathrm{O}}^{best}$ as the SQP initial guess, that is, ${q}_{0}={q}_{\mathrm{A}\mathrm{C}\mathrm{O}}^{best}$. Here, ${q}_{0}$ is the starting point of the SQP stage. The ACO output is used only for initialization and does not replace the common continuity reference ${q}_{t}^{ref}$ in the objective function.

**Step 5. Solve the SQP quadratic subproblem:** Starting from ${q}_{0}$, solve the SQP quadratic subproblem in (34) for the common objective function in (30), subject to the linearized joint-limit constraints in (37)–(38). Then update the joint vector according to (35).

**Step 6. Repeat the SQP updates until convergence:** Continue solving the SQP subproblem and updating the joint vector until the convergence condition is satisfied or the maximum SQP iteration count is reached. In this stage, SQP acts as a local continuous refinement step around the promising region identified by ACO.

**Step 7. Stop the SQP stage and return the final hybrid solution:** Terminate the SQP stage when the desired convergence condition is satisfied or when the maximum SQP iteration count is reached. The final solution of the hybrid ACO–SQP method is then given by ${q}_{\mathrm{A}\mathrm{S}}^{*}={q}^{*},$ where ${q}^{*}$ denotes the converged SQP solution obtained from the initial point ${q}_{0}={q}_{\mathrm{A}\mathrm{C}\mathrm{O}}^{best}$. Here, ${q}_{\mathrm{A}\mathrm{S}}^{*}$ is the final solution produced by the ACO–SQP hybrid method.

To ensure a fair comparison, in the ACO-SQP method the ACO output is used only to initialize SQP; it does not replace the common continuity reference ${{\boldsymbol{q}}}_{t}^{ref}$​ or alter the common objective function ${J}_{t}(q)$, desired Cartesian pose, or joint-limit constraints used by all compared methods.

**Main idea of the proposed ACO–SQP method:** The proposed ACO–SQP framework uses ACO not as the final optimizer, but as a global seed generator for subsequent continuous SQP refinement. In this way, the stochastic and diversity-preserving behavior of ACO explores the discretized inverse-kinematics search space and identifies a promising candidate solution or basin of attraction, while SQP performs fast deterministic local refinement from that candidate under the common objective function and joint-limit constraints. This coupling reduces the initialization sensitivity of standalone SQP and makes the overall search more robust in multimodal inverse-kinematics landscapes, while preserving the fast local convergence properties of SQP.

**Justification for the order ACO → SQP:** The order ACO → SQP is selected because it establishes an effective progression from global exploration to local continuous refinement. ACO is particularly suitable for the first stage because it can search a large nonconvex and multimodal joint space without requiring derivative information and can provide a good-quality initial solution even when multiple inverse-kinematics branches exist. Once such a promising candidate has been identified, SQP becomes more effective as the second stage because it can rapidly refine that candidate to a locally optimized feasible solution under the imposed constraints. If SQP were used as the first stage, its convergence would depend strongly on the initial guess and it could converge quickly to a suboptimal local minimum. By contrast, placing ACO after SQP would reintroduce a stochastic global search after deterministic local refinement, which would weaken the role of SQP as a final polishing step. Accordingly, the sequence ACO → SQP is adopted as the most suitable arrangement for combining broad search coverage with efficient local convergence.

*H. Hybrid B&B–SQP mode*

**Fig. 10:**
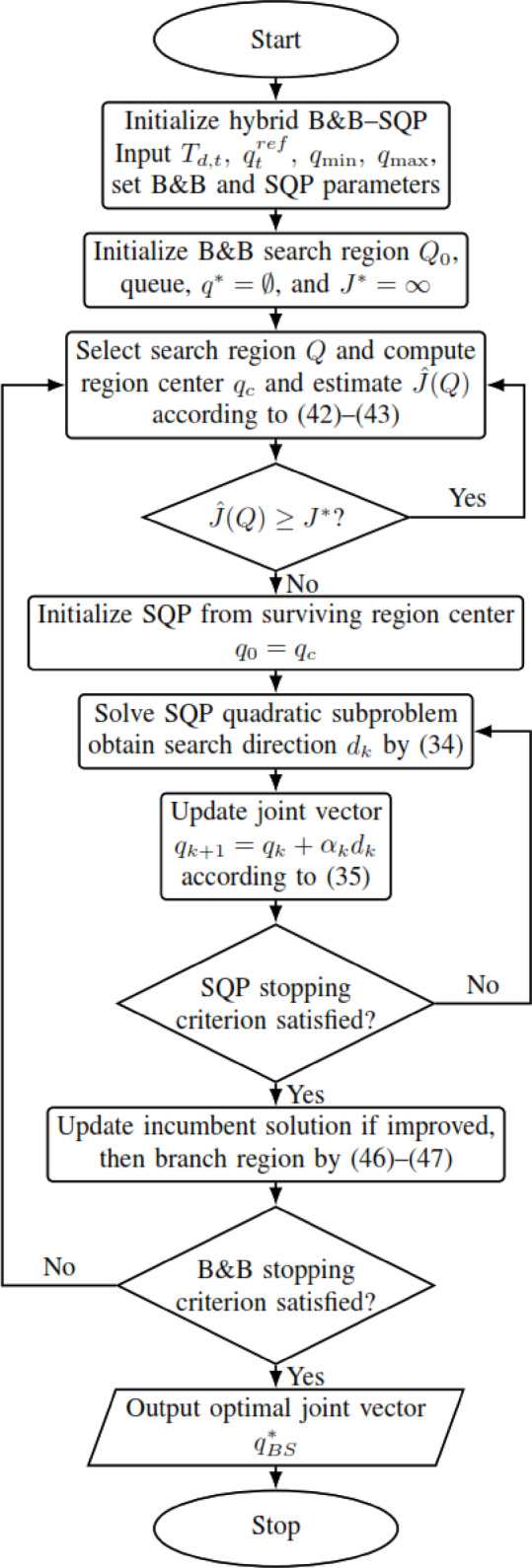
B&B-SQP flowchart.

This method applies a heuristic bounded-search strategy for 6-DOF inverse kinematics by integrating a B&B-style partition-and-screen procedure with Sequential Quadratic Programming (SQP). The B&B stage partitions the admissible joint space and uses the center-point objective estimate in (43) to prioritize and heuristically prune regions. SQP is then used as a local continuous optimizer to refine promising candidates under the same common objective function and joint-limit constraints. Because the regional screening criterion is heuristic and not based on a mathematically valid lower bound, the method is not claimed to be a rigorous global optimizer. Instead, it provides broader bounded search coverage than standalone SQP while preserving efficient local refinement. To ensure a fair comparison, this hybrid method uses the same desired Cartesian pose ${T}_{d,t}$​, the same continuity reference ${{\boldsymbol{q}}}_{t}^{ref}$​, the same joint-limit constraints, and the same common objective function ${J}_{t}(q)$ as the other compared methods; only the search procedure differs. The main steps of the method are summarized below, and the corresponding flowchart is shown in Fig. 10:

**Step 1. Define the common inverse-kinematics optimization problem:** Same as Step 1 of the proposed ACO-SQP method.

**Step 2. Initialize the B&B stage over the full admissible joint space:** Initialize the B&B search region by the hyper-rectangle ${Q}_{0}$ in (41). Also initialize the incumbent best solution ${q}^{*}$ and the corresponding best objective value ${J}^{*}$. Insert the initial region ${Q}_{0}$ into a priority queue ordered by the regional objective estimate $\widehat{J}\left(Q\right)$ defined in (43).

**Step 3. Select a search region and evaluate its center-point estimate:** Remove one region $Q$ from the queue, compute its center point ${q}_{c}$ according to (42), and evaluate the regional objective estimate $\widehat{J}\left(Q\right)$ using (43). Here, ${q}_{c}$ is the center point of the current B&B search region and is used only for regional evaluation and local initialization.

**Step 4. Apply the B&B heuristic pruning rule:** If the regional estimate satisfies $\widehat{J}\left(Q\right)\ge {J}^{*}$ according to the pruning rule in (45), prune the region and continue to the next queued region. Otherwise, retain the region for local SQP refinement.

**Step 5. Initialize the SQP stage from the surviving B&B region center:** For each region that survives pruning, use the corresponding center point ${q}_{c}$ as the SQP initial guess, that is, ${q}_{0}={q}_{c}.$ Here, ${q}_{0}$ is the starting point of the SQP stage for that regional search box.

**Step 6. Solve the SQP quadratic subproblem from the selected region center:** Starting from ${q}_{0}$, solve the SQP quadratic subproblem in (34) for the common objective function in (30), subject to the linearized joint-limit constraints in (37)–(38). Then update the joint vector according to (35) until a refined local solution ${q}_{\mathrm{S}\mathrm{Q}\mathrm{P}}$ is obtained.

**Step 7. Update the incumbent best solution:** Evaluate the refined solution using the common objective function in (30). If ${J}_{t}\left({q}_{\mathrm{S}\mathrm{Q}\mathrm{P}}\right)<{J}^{*},$ update the incumbent solution and objective value accordingly. In this way, the B&B stage provides regional search coverage, while the SQP stage provides continuous local refinement.

**Step 8. Branch the current region and continue the B&B search:** For the current region, select the branching variable according to the largest-interval rule in (46), and split that interval into two child regions according to (47). Insert the child regions into the priority queue and continue the search.

**Step 9. Stop the hybrid procedure and return the final solution:** Terminate the algorithm when the search queue becomes empty, when the maximum number of explored nodes is reached, or when the interval width falls below the minimum threshold. The final solution of the hybrid B&B–SQP method is then given by ${q}_{\mathrm{B}\mathrm{S}}^{*}={q}^{*}.$ Here, ${q}_{\mathrm{B}\mathrm{S}}^{*}$ is the final solution produced by the B&B–SQP hybrid method.

To ensure a fair comparison, in the B&B-SQP method the B&B region center ${q}_{c}$ is used only for regional evaluation and to initialize local SQP refinement; it does not replace the common continuity reference ${{\boldsymbol{q}}}_{t}^{ref}$​ or alter the common objective function ${J}_{t}(q)$ used for all compared methods.

**Main idea of the proposed B&B–SQP method:** The proposed B&B–SQP framework uses B&B not as a final exact global optimizer, but as a structured bounded-search mechanism that identifies promising continuous subregions for subsequent SQP refinement. In this way, the partitioning and heuristic pruning behavior of B&B narrows the admissible inverse-kinematics search space to regions that are more likely to contain good feasible solutions, while SQP performs deterministic continuous local refinement from representative candidates of those regions under the common objective function and joint-limit constraints. This coupling reduces the initialization sensitivity of standalone SQP, provides broader search coverage than a purely local method, and keeps the final refinement computationally efficient.

**Justification for the order B&B → SQP:** The order B&B → SQP is selected because it provides an effective progression from bounded regional search to fast local continuous refinement. B&B is particularly suitable for the first stage because it can systematically partition the admissible joint space, evaluate promising regions, and heuristically discard regions that are unlikely to improve the current incumbent solution. Once such promising regions have been identified, SQP becomes more effective as the second stage because it can refine the center or representative candidate of each surviving region to a locally optimized feasible solution. If SQP were used as the first stage, the search would again become strongly dependent on initialization and could terminate in a poor local basin before broader regional exploration had occurred. By contrast, placing B&B after SQP would require a bounded partition search after local optimization had already been completed, which would reduce the efficiency of the hybrid structure and duplicate search effort. Accordingly, the sequence B&B → SQP is adopted as the most suitable arrangement for combining broader bounded search coverage with efficient final local refinement.

*I. Hybrid ACO–B&B–SQP mode*

**Fig. 11:**
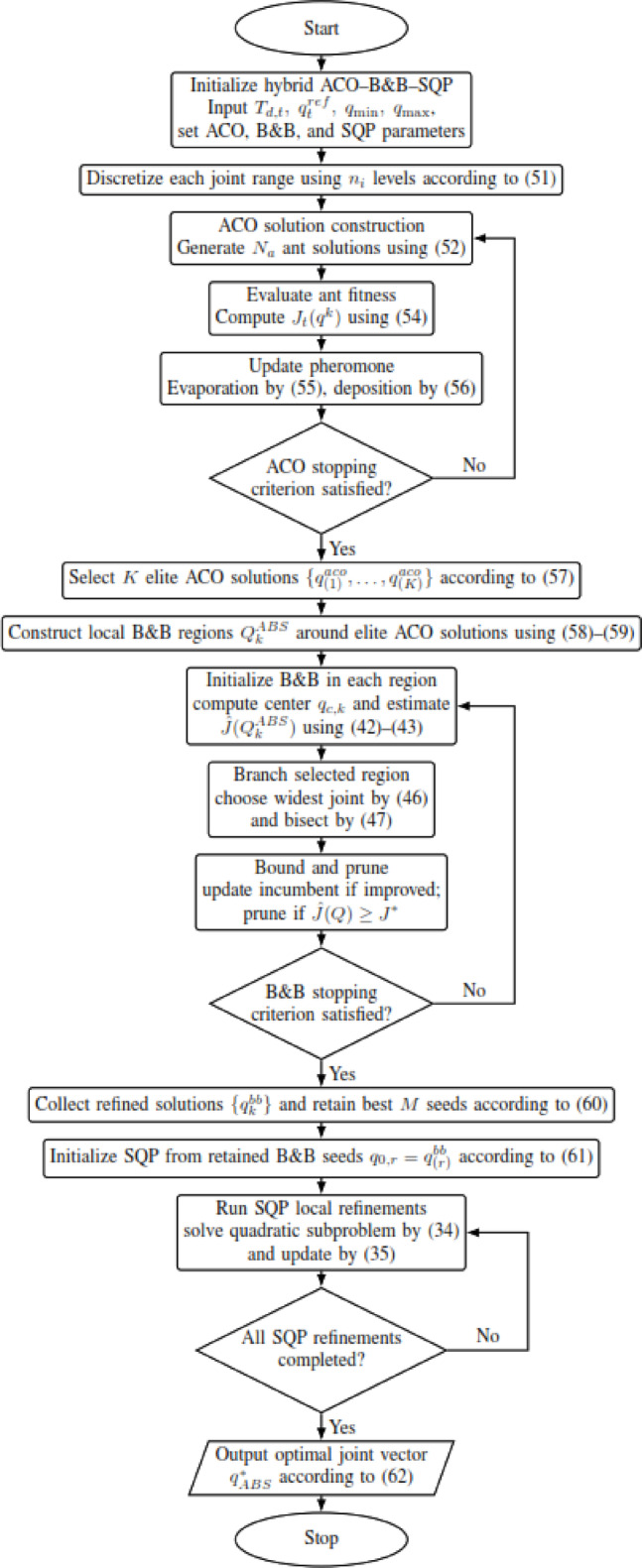
ACO-B&B-SQP flowchart.

The proposed hybrid ACO–B&B–SQP method is a three-stage multi-resolution optimizer. ACO first performs coarse global exploration on the discretized joint space. B&B then contracts the search into a small number of promising continuous subregions. Finally, SQP performs continuous local refinement inside and around those refined regions to obtain the final IK solution. For fairness, this hybrid method uses the same desired Cartesian pose ${T}_{d,t}$​, the same continuity reference ${{\boldsymbol{q}}}_{t}^{ref}$, the same joint-limit constraints, and the same objective function ${J}_{t}(q)$ as the other compared methods; only the search procedure differs. The mathematical steps of the proposed method, together with the corresponding flowchart, are shown in Fig. 11.

**Step 1. Define the common inverse-kinematics optimization problem:** Same as Step 1 of the proposed ACO-SQP method.

**Step 2. Run the ACO stage over the full joint space:** Initialize the ACO parameters and discretize each admissible joint interval according to (51). Then construct ant solutions using the continuity-based heuristic in (50), the transition probabilities in (52), the fitness function in (54), and the pheromone-update rules in (55) and (56). For each candidate solution, evaluate the forward kinematics using (23), compute the quaternion-based orientation error using (28), and evaluate the common objective value using (54).

**Step 3. Sort the feasible ACO solutions and retain the best** $K$ **elite solutions:** After the ACO stage terminates, sort the feasible ACO solutions and retain the best $K$ of them as the elite set for the B&B stage:

57 $${E}_{K}^{\mathrm{A}\mathrm{B}\mathrm{S}}=\{{q}_{\left(1\right)}^{aco},{q}_{\left(2\right)}^{aco},\dots ,{q}_{\left(K\right)}^{aco}\}, {J}_{t}\left({q}_{\left(1\right)}^{aco}\right)\le {J}_{t}\left({q}_{\left(2\right)}^{aco}\right)\le \cdots \le {J}_{t}\left({q}_{\left(K\right)}^{aco}\right)$$

Here, $K$ is the number of elite ACO solutions retained for regional B&B refinement, ${E}_{K}^{\mathrm{A}\mathrm{B}\mathrm{S}}$ is the elite ACO set, and ${q}_{\left(k\right)}^{aco}$ is the $k$-th best ACO solution after sorting.

**Step 4. Define the local B&B refinement width for each joint:** For each joint $i$, define the half-width of the local B&B refinement interval as

58 $${\delta}_{i}^{\mathrm{A}\mathrm{B}\mathrm{S}}={\gamma}_{\mathrm{A}\mathrm{B}\mathrm{S}}\left({\theta}_{i}^{max}-{\theta}_{i}^{min}\right)$$

Here, ${\gamma}_{\mathrm{A}\mathrm{B}\mathrm{S}}$ is the local-region scaling factor for the B&B stage of the ACO–B&B–SQP method, with $0<{\gamma}_{\mathrm{A}\mathrm{B}\mathrm{S}}\le 0.5$, and ${\delta}_{i}^{\mathrm{A}\mathrm{B}\mathrm{S}}$ is the half-width of the B&B regional interval around joint $i$.

**Step 5. Construct a bounded B&B refinement region around each elite ACO solution:** Using the interval width defined above, construct a local B&B search region around each elite ACO solution ${q}_{\left(k\right)}^{aco}$ as

59 $${Q}_{k}^{\mathrm{A}\mathrm{B}\mathrm{S}}=\prod_{i=1}^{6}\left[\mathrm{m}\mathrm{a}\mathrm{x}\left({\theta}_{i}^{min},{\theta}_{i,\left(k\right)}^{aco}-{\delta}_{i}^{\mathrm{A}\mathrm{B}\mathrm{S}}\right),\mathrm{m}\mathrm{i}\mathrm{n}\left({\theta}_{i}^{max},{\theta}_{i,\left(k\right)}^{aco}+{\delta}_{i}^{\mathrm{A}\mathrm{B}\mathrm{S}}\right)\right]$$

Here, ${Q}_{k}^{\mathrm{A}\mathrm{B}\mathrm{S}}$ is the B&B refinement region generated from the $k$-th elite ACO solution, and ${\theta}_{i,\left(k\right)}^{aco}$ is the value of joint $i$ in that elite ACO solution.

**Step 6. Apply B&B independently inside each regional search box:** Next, apply B&B independently within each region ${Q}_{k}^{\mathrm{A}\mathrm{B}\mathrm{S}}$. For each region, compute the center point ${q}_{c,k}$ according to (42), evaluate the regional objective estimate $\widehat{J}\left({Q}_{k}^{\mathrm{A}\mathrm{B}\mathrm{S}}\right)$ using (43), and apply the pruning rule in (45). Then continue the bounded search using the branching rule in (46) and the interval split in (47). Let ${q}_{k}^{bb}$ denote the best feasible solution found by the $k$-th regional B&B search, for $k=1,2,\dots ,K$.

**Step 7. Rank the B&B-refined solutions and retain the best regional seeds for SQP:** After all regional B&B searches are completed, sort the refined B&B outputs according to the common objective value and retain the best $M$ of them as the seed set for the final SQP stage:

60 $${B}_{M}^{\mathrm{A}\mathrm{B}\mathrm{S}}=\{{q}_{\left(1\right)}^{bb},{q}_{\left(2\right)}^{bb},\dots ,{q}_{\left(M\right)}^{bb}\}, {J}_{t}\left({q}_{\left(1\right)}^{bb}\right)\le {J}_{t}\left({q}_{\left(2\right)}^{bb}\right)\le \cdots \le {J}_{t}\left({q}_{\left(M\right)}^{bb}\right)$$

Here, $M$ is the number of best B&B-refined solutions used to initialize the SQP stage, with $1\le M\le K$, ${B}_{M}^{\mathrm{A}\mathrm{B}\mathrm{S}}$ is the refined seed set, and ${q}_{\left(r\right)}^{bb}$ is the $r$-th best B&B-refined solution after sorting.

**Step 8. Initialize the SQP stage from the retained B&B-refined solutions:** For each retained B&B-refined seed, use that solution as the initial point of a local SQP refinement, that is,

61 $${q}_{0,r}={q}_{\left(r\right)}^{bb}, r=1,2,\dots ,M.$$

Here, ${q}_{0,r}$ is the initial SQP point generated from the $r$-th retained B&B-refined solution.

**Step 9. Run the SQP refinements and compare the final local solutions:** Starting from each initial point ${q}_{0,r}$, solve the SQP quadratic subproblem in (34) for the common objective function in (30), subject to the linearized joint-limit constraints in (37)–(38), and update the joint vector according to (35) until convergence to a refined local solution ${q}_{\mathrm{S}\mathrm{Q}\mathrm{P},r}^{*}$. After all SQP refinements are completed, compare the final refined candidates and retain the best one according to the common objective value.

**Step 10. Stop the hybrid procedure and return the final solution:** Terminate the hybrid procedure when the ACO stopping condition is satisfied, the B&B stopping condition is satisfied, and all SQP refinements have converged. The final solution of the hybrid ACO–B&B–SQP method is then given by

62 $${q}_{\mathrm{A}\mathrm{B}\mathrm{S}}^{*}=\mathrm{a}\mathrm{r}\mathrm{g}\underset{r\in \{1,\dots ,M\}}{\mathrm{m}\mathrm{i}\mathrm{n}}{J}_{t}\left({q}_{\mathrm{S}\mathrm{Q}\mathrm{P},r}^{*}\right)$$

Here, ${q}_{\mathrm{A}\mathrm{B}\mathrm{S}}^{*}$ is the final solution produced by the ACO–B&B–SQP hybrid method, and ${q}_{\mathrm{S}\mathrm{Q}\mathrm{P},r}^{*}$ is the converged SQP solution obtained from the $r$-th retained B&B-refined seed.

To ensure a fair comparison, in the ACO-B&B-SQP method the ACO candidate solutions, the B&B refinement regions, and the SQP initializations are internal search variables only; they do not replace the common reference configuration ${{\boldsymbol{q}}}_{t}^{ref}$​ or alter the common objective function ${J}_{t}\left(q\right)$, the desired Cartesian pose, or the joint-limit constraints shared by all compared methods.

**Main idea of the proposed ACO–B&B–SQP method:** The proposed ACO–B&B–SQP framework is a three-stage hybrid strategy in which ACO performs coarse global exploration, B&B performs structured regional contraction, and SQP performs final continuous local refinement. In this way, the stochastic and diversity-preserving behavior of ACO identifies several high-quality inverse-kinematics basins of attraction over the discretized joint space, B&B then refines the search deterministically only inside bounded regions generated from those elite ACO solutions, and SQP finally polishes the best regional candidates under the common objective function and joint-limit constraints. This coupling reduces unnecessary full-domain search, makes the overall optimization more computationally focused, and combines the complementary strengths of broad exploration, deterministic bounded refinement, and fast local convergence.

**Justification for the order ACO → B&B → SQP:** The order ACO → B&B → SQP is selected because it establishes a natural progression from coarse global exploration to structured regional refinement and finally to local continuous polishing. ACO is most suitable for the first stage because it can explore a large multimodal search space without derivative information and identify several promising candidate regions rather than a single local seed. B&B is then more effective as the second stage because, once these promising regions are known, it can contract the search deterministically inside a much smaller bounded domain and prune subregions that are unlikely to improve the incumbent solution. SQP is most suitable as the final stage because, after the search has already been concentrated into high-quality local neighborhoods, it can exploit its fast local convergence to produce a refined feasible solution efficiently. If B&B were placed before ACO, it would need to partition and screen the full initial domain, which would substantially increase the search complexity. If SQP were placed before ACO or B&B, the procedure would become too dependent on a local initial guess and could lose the benefit of broad multimodal exploration. Likewise, placing ACO after B&B or SQP would reintroduce stochastic global sampling after deterministic refinement, which would weaken the efficiency of the final optimization stage. Accordingly, the sequence ACO → B&B → SQP is adopted as the most suitable arrangement for combining global coverage, bounded-search efficiency, and accurate final local refinement.

*J. Hyperparameter selection and sensitivity analysis*

To improve reproducibility, the main algorithmic hyperparameters of ACO, B&B, and SQP were selected before the final comparative experiments and then kept fixed for all reported runs and for both test trajectories. Parameter selection was performed on a development set consisting of representative target poses and short trajectory segments covering both 2D and 3D motion conditions. The objective of tuning was not to optimize each method separately for a single trajectory, but to identify stable settings that provide a reasonable runtime-accuracy trade-off under the common IK formulation used throughout this study.

For ACO, the tuned parameters include the pheromone exponent $\alpha$, heuristic exponent $\beta$, evaporation rate $\rho$, number of ants ${N}_{a}$​, maximum number of iterations ${N}_{max}$​, stall limit ${N}_{stall}$​, objective-improvement tolerance ${\upvarepsilon}_{J}$​, pheromone scaling factor ${Q}_{ph}$​, and the joint discretization level ${n}_{i}$​. The final values used in all experiments were $\alpha =1$, $\beta =2$, $\rho =0.1$, ${N}_{a}=30$, ${N}_{max}=100$, ${N}_{stall}=15$, $\upvarepsilon ={10}^{-6}$, ${\upvarepsilon}_{J}={10}^{-6}$, ${Q}_{ph}=1$, ${n}_{i}=51$. These values were chosen because they provided a consistent balance between exploration, convergence stability, and computational cost across the tested cases. In particular, $\beta =2$ gave a useful continuity bias toward the reference configuration without overwhelming pheromone-guided exploration, while ${n}_{i}=51$ provided a practically acceptable discretization density for generating candidate seeds for the hybrid methods.

For B&B, the main parameters are the maximum allowed number of explored nodes ${N}_{node,max}$​, the minimum interval width $\Delta {\theta}_{min}$​, and the queue ordering based on the regional center-point objective estimate $\widehat{J}\left(\mathcal{Q}\right)$. The final settings used in all experiments were ${N}_{node,max}={10}^{4}$, $\Delta {\theta}_{min}={10}^{-3}$ rad, with branching performed along the joint dimension having the largest interval width. These settings were selected as a practical compromise between search coverage and runtime. Larger node budgets increased coverage but also caused substantial growth in computation time, whereas smaller budgets could terminate the bounded search prematurely. Similarly, a smaller $\Delta {\theta}_{min}$​ provided finer partition refinement but increased search cost with diminishing practical benefit under the present study.

For SQP, the implementation used a fixed local optimization configuration across all experiments, including the same Hessian update strategy, stopping tolerances, and iteration limits. In the revised manuscript, these settings are now reported explicitly as follows: a BFGS quasi-Newton Hessian approximation, maximum iteration limit of 100, step tolerance of ${10}^{-6}$, optimality tolerance of ${10}^{-6}$, and constraint tolerance of ${10}^{-6}$. The same SQP configuration was used for standalone SQP and for the SQP stage inside the hybrid methods. This fixed configuration was adopted deliberately so that differences between SQP-based variants reflect the effect of initialization and search strategy rather than changes in the local solver itself.

In addition to reporting the final values, a local sensitivity analysis was carried out by perturbing the main parameters around their selected settings while holding the remaining parameters fixed. For ACO, $\rho$ was varied by ± 50%, $\beta$ by ± 1, the number of ants by ± 10, and the discretization level ${n}_{i}$ was checked at 31, 41, 51, and 61. For B&B, sensitivity was examined with respect to ${N}_{node,max}$​ and $\Delta {\theta}_{min}$​. For SQP, sensitivity was examined with respect to the Hessian update strategy and stopping tolerances. The purpose of this analysis was to verify whether the relative behavior of the methods changed substantially under moderate parameter perturbations.

In particular, the discretization study over ${n}_{i}=\left\{\mathrm{31,41,51,61}\right\}$ suggested that ${n}_{i}=51$ provided a reasonable middle ground: lower values reduced seed quality in some cases, whereas higher values increased runtime without changing the main qualitative conclusions of the study.

The local sensitivity study indicated that the reported qualitative conclusions were reasonably stable under moderate parameter variations, although, as expected, the runtime and final path error of the stochastic and search-based methods were influenced by the exploration-related parameters. Increasing the ACO discretization level improved seed quality at the cost of higher runtime, while tighter B&B stopping criteria increased search effort with limited additional benefit beyond the selected setting. For SQP, tighter stopping tolerances slightly improved local refinement in some cases but increased runtime, whereas looser tolerances reduced runtime at the risk of earlier termination. Therefore, the final parameter values were retained as a balanced configuration for the unified comparative evaluation reported in this work.

A summary of the tested parameter ranges, final selected values, and the observed runtime/error trends is provided in Table 6.

**Table 6:** Hyperparameter tuning ranges, final values, and qualitative sensitivity trends.

Method	Parameter	Tested range / options	Final value	Main observed effect
ACO	$\alpha$	0.5, 1, 2	1	Higher values increased pheromone dominance and reduced early exploration
	$\beta$	1, 2, 3	2	Higher values increased heuristic bias toward continuity
	$\rho$	0.05, 0.10, 0.15	0.10	Larger $\rho$ accelerated forgetting; smaller $\rho$ slowed adaptation
	${N}_{a}$	20, 30, 40	30	More ants improved exploration but increased runtime
	${N}_{max}$	75, 100, 125	100	More iterations improved convergence but increased runtime
	${N}_{stall}$	10, 15, 20	15	Larger values delayed termination and slightly increased runtime
	${n}_{i}$	31, 41, 51, 61	51	Finer discretization improved seed resolution but expanded search cost
B&B	${N}_{node,max}$	$5*{10}^{3}$, ${10}^{4}$, 2 $*{10}^{4}$	${10}^{4}$	Larger values increased coverage and runtime
	$\Delta {\theta}_{min}$	${10}^{-2}$, ${10}^{-3}$, ${10}^{-4}$ rad	${10}^{-3}$ rad	Smaller values refined search but increased runtime
	Queue ordering	FIFO, depth-priority, $\widehat{J}\left(\mathcal{Q}\right)$-priority	$\widehat{J}\left(\mathcal{Q}\right)$-priority	Objective-based ordering improved practical search focus
SQP	Hessian update	BFGS, SR1	BFGS	BFGS gave more stable local convergence
	Max iterations	50, 100, 150	100	Larger values allowed more refinement but increased runtime
	Step tolerance	${10}^{-5}$, ${10}^{-6}$,${10}^{-7}$	${10}^{-6}$	Tighter tolerance increased runtime with limited added benefit
	Optimality tolerance	${10}^{-5}$, ${10}^{-6}$,${10}^{-7}$	${10}^{-6}$	Tighter tolerance improved refinement slightly but slowed convergence
	Constraint tolerance	${10}^{-5}$, ${10}^{-6}$,${10}^{-7}$	${10}^{-6}$	Tighter tolerance improved feasibility control but increased solve time

*K. Convergence, stopping criteria, and optimality remarks*

Because the inverse-kinematics problem defined in (29)–(30) is nonlinear, nonconvex, and subject to joint-limit constraints, a general guarantee of global optimality is not available for the six methods considered in this study. Accordingly, the present work does not claim theoretical global convergence or global optimality for SQP, ACO, the present B&B implementation, ACO-SQP, B&B-SQP, or the three-stage ACO-B&B-SQP method. Instead, all methods are compared empirically under the same robot model, common objective function, joint-limit constraints, trajectories, and evaluation metrics, while each method uses its own explicitly reported stopping rules.

For **SQP**, convergence is interpreted in the local optimization sense. In the present nonconvex IK formulation, the converged solution may depend on the initial guess and may correspond to a local minimum rather than a globally optimal one. Therefore, SQP is treated here as a local constrained optimizer within the common IK framework.

For **ACO**, convergence is interpreted in the practical metaheuristic sense through improvement of the best-so-far feasible solution across iterations. In the present implementation, the ACO stage terminates when either the maximum number of iterations is reached or when the improvement in the best objective value remains below a prescribed tolerance for a specified number of consecutive iterations. These stopping rules provide a reproducible numerical definition of practical convergence, but they do not constitute a proof of convergence to the global optimum.

For **B&B**, the search proceeds through recursive partitioning of the admissible joint-space domain and heuristic pruning of regions. However, in the present implementation, the regional quantity $\widehat{J}\left(Q\right)$ is computed from the objective value at the region center and is used only as a heuristic regional estimate, not as a mathematically valid lower bound derived from a convex relaxation or interval analysis of the nonlinear forward-kinematics-based objective. Therefore, the implemented B&B stage should be interpreted as a structured bounded search with heuristic pruning rather than as an exact global branch-and-bound algorithm with certified global optimality. The B&B search terminates when the maximum explored-node limit is reached, when the maximum interval width falls below the prescribed minimum threshold, or when the search queue becomes empty.

The **hybrid methods** inherit the strengths and limitations of their constituent stages. In **ACO-SQP**, ACO is used to generate a promising global seed and SQP then performs continuous local refinement. In **B&B-SQP**, B&B provides bounded regional search and SQP refines the surviving regional candidates. In **ACO-B&B-SQP**, the optimization proceeds sequentially from coarse global exploration to bounded regional contraction and finally to local continuous polishing. These hybrid procedures are not claimed to be globally convergent in the theoretical sense; rather, they are designed to improve empirical robustness and solution quality by combining complementary search mechanisms. For the hybrid solvers, practical convergence is defined by completion of the preceding search stages together with termination of the SQP refinement stage according to its prescribed stopping rule; for the ACO-B&B-SQP method, this means completion of all retained SQP refinements before selecting the best final candidate.

From an optimization perspective, the present study is therefore positioned as a **controlled empirical comparative evaluation** under a unified IK framework, rather than as a proof of global convergence or global optimality. The reported results should be interpreted accordingly: they show comparative differences in runtime, mean geometric path error, within-run dispersion, and run-to-run variability under the specified experimental conditions, while formal global convergence guarantees remain outside the scope of the current work.

## Results and discussion

**Fig. 12:**
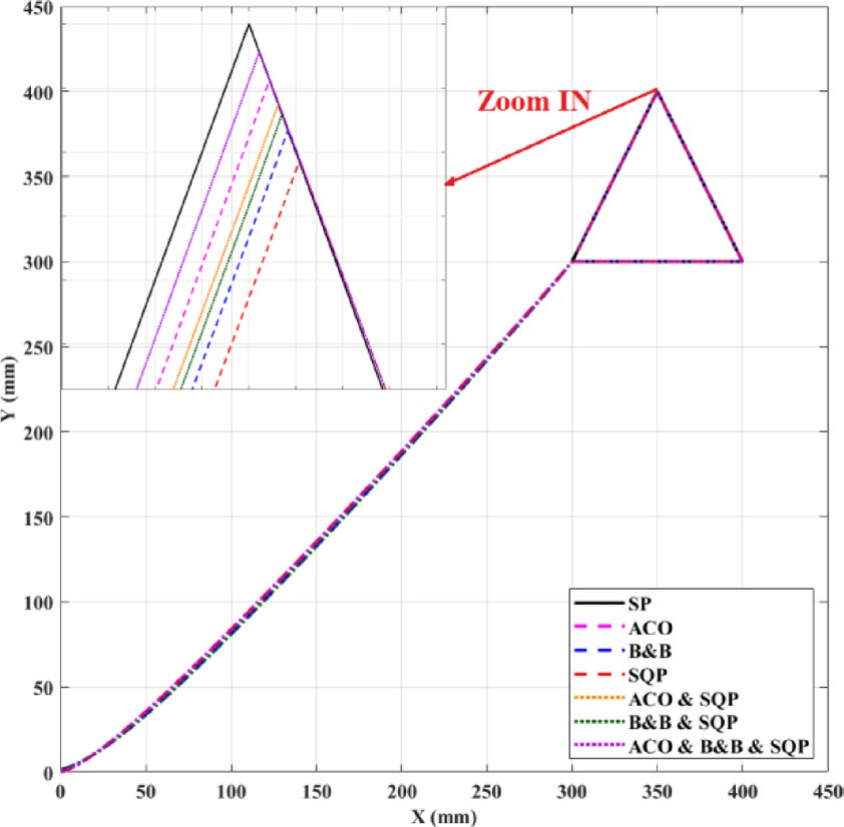
2D triangle (mm).

**Fig. 13:**
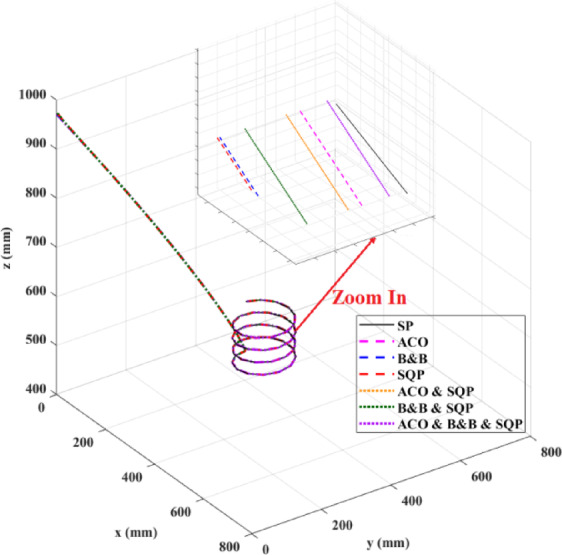
3D helix (mm).

**Fig. 14:**
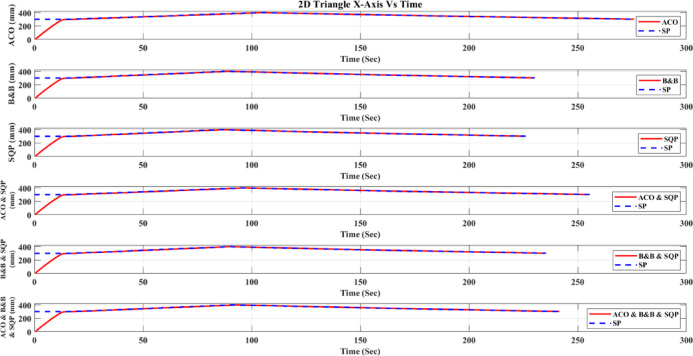
2D Triangle X-Axis (mm) vs Time.

**Fig. 15:**
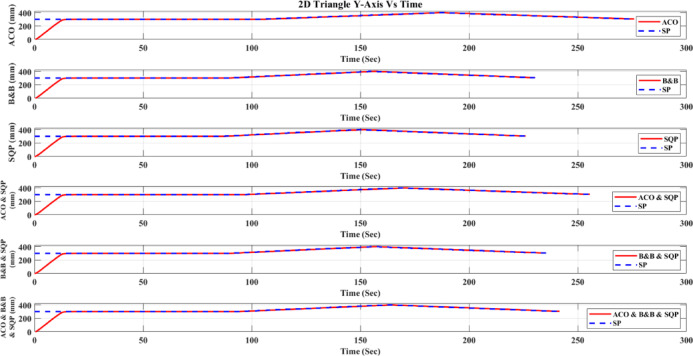
2D Triangle Y-Axis (mm) vs Time.

**Fig. 16:**
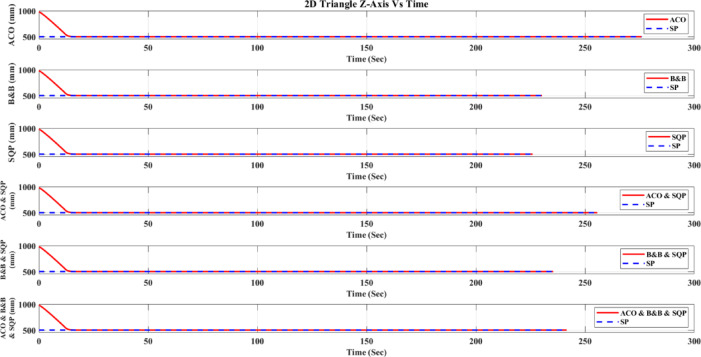
2D Triangle Z-Axis (mm) Vs Time.

**Fig. 17:**
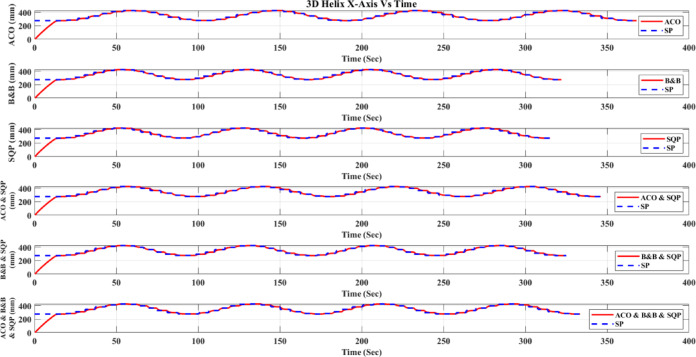
3D Helix X-Axis (mm) vs Time.

**Fig. 18:**
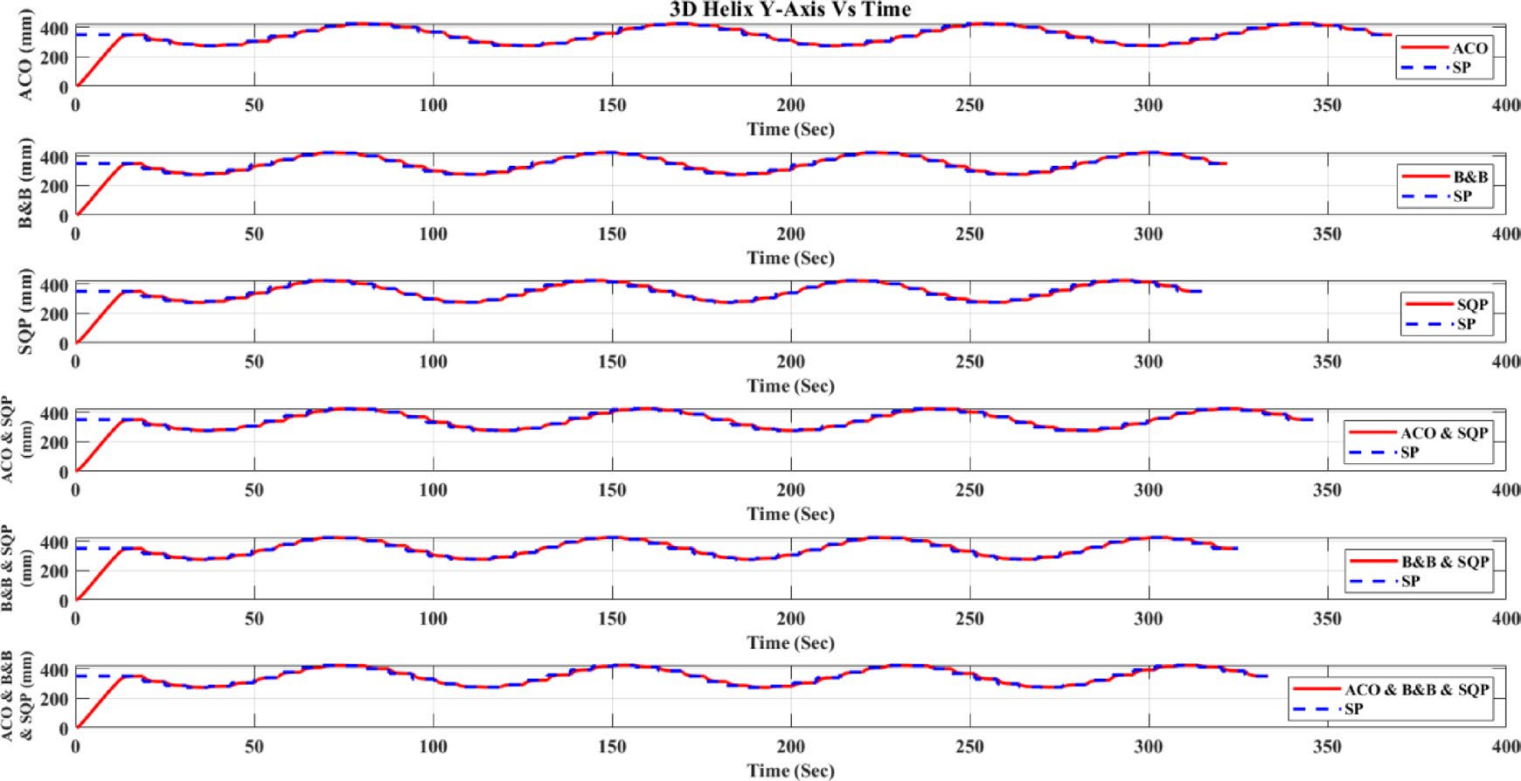
3D Helix Y-Axis (mm) vs Time.

**Fig. 19:**
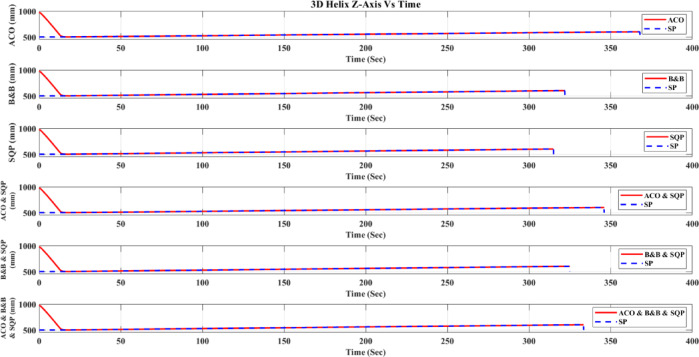
3D Helix Z-Axis (mm) vs Time.

Table 7 compares the tested optimization methods—SQP as a continuous optimizer, B&B and ACO as structured/discrete search methods, and the hybrid combinations ACO-SQP, B&B-SQP, and ACO-B&B-SQP—on the 2D triangle and 3D helix trajectories shown in Figs. 12, 13, 14, 15, 16, 17, 18 and 19. For all methods, the nominal reference trajectory is identical and is defined by the same 51 reference set-points. During robot execution in ACE, however, the realized end-effector motion is recorded as a dense sequence of actual samples. The quantity $n$ reported in Table 7 denotes this logged sample count of the executed path, not the number of reference set-points. Because the executed motion duration, interpolation progression, and controller-generated motion profile may differ slightly from one method to another, the number of logged actual samples $n$ is not identical across methods, even though the same nominal reference path is used in every case.

**Table 7:** Results analysis.

Shape	Optimization method	Trajectory execution time (Sec)	Mean Path Error (mm)	Std. Dev. (mm)	Std. Dev. across 20 runs (mm)	Actual path point ($n$)
2D Triangle	ACO	275.9267	2.07882	0.2265443	0.22714	293,292
	B&B	230.2791	2.48478	0.2464098	0.24952	245,363
	SQP	226.0294	2.53194	0.2501688	0.25571	242,215
	Hybrid ACO with SQP	255.601	2.23866	0.23609	0.23853	273,978
	Hybrid B&B with SQP	235.4096	2.43397	0.2455888	0.24525	251,956
	Hybrid ACO-B&B-SQP	241.6114	1.84441	0.21402	0.21621	257,257
3D Helix	ACO	368.0058	2.05002	0.201811	0.20621	393,875
	B&B	321.982	2.3318	0.2142338	0.21482	346,431
	SQP	314.969	2.40781	0.2202045	0.22263	335,975
	Hybrid ACO with SQP	346.0716	2.18938	0.2106105	0.21135	369,752
	Hybrid B&B with SQP	324.9812	2.32794	0.2167213	0.21921	347,709
	Hybrid ACO-B&B-SQP	333.4411	1.87155	0.193358	0.20031	357,799

In Table 7, **Std. Dev. (mm)** is computed by (65) and represents the within-run standard deviation of the instantaneous geometric path error over the $n$ logged actual samples, whereas **Std. Dev. across 20 runs (mm)** is computed by (67) and represents the run-to-run standard deviation of the run-level mean path error over 20 repeated runs. The quantity **Actual path point (**$n$**)** denotes the number of densely logged end-effector samples recorded during execution of one optimized trajectory in ACE. It is distinct from the 51-reference set-points, which are fixed and identical for all methods. Differences in $n$ arise because the executed trajectories may differ slightly in total duration, interpolation progression, and motion profile, which changes how many samples are recorded by the logging system. Therefore, $n$ reflects measurement density during execution rather than a difference in the commanded reference path. The same path-generation procedure, robot model, and error-evaluation script were used for all methods.

*A. Experimental platform*

To clarify the experimental platform, all tests were conducted on an Omron Adept Viper s650 six-axis industrial robot, controlled through the Omron eMB-60R robot controller and programmed via the Automation Control Environment (ACE), while the optimization routines were executed on a Windows 11 PC with an Intel Core i7-7700HQ processor (2.80 GHz) and 16 GB RAM. Omron documentation lists the Viper s650 in a “Viper with eMB-60R” configuration, and related Viper systems are also associated with the SmartController EX generation of control hardware. The implementations of SQP, B&B, ACO, and the hybrid methods were all developed and executed within the same ACE–MATLAB framework, using identical robot model parameters, constraints, and objective definitions. The reported execution time corresponds to the total wall-clock time required to generate the optimized solution for one complete trajectory, rather than the time per sample or per set-point.

Trajectory execution Time (sec) is the wall-clock runtime for one complete optimization run for one full trajectory, i.e., the total time required to compute the entire optimized trajectory solution (including objective/constraint evaluations and IK calls as applicable). Therefore, the reported time is per trajectory optimization, not time per sample and not time per set point.

**Performance metrics:** Performance is evaluated using Trajectory execution time (sec) and mean path error (mm). Path error measures the robot’s geometric deviation from the ideal path, independent of timing, rather than a time-aligned correspondence.

To avoid ambiguity, the mean path error reported in this work is a geometric path-deviation measure computed from the logged actual execution trace relative to the same nominal reference path. It is not intended as a strict time-aligned servo-tracking metric. Accordingly, differences in the logged sample count $n$ reflect differences in execution duration and sampling density rather than differences in the number of commanded reference points. Under this evaluation protocol, variation in $n$ mainly affects the granularity with which the executed path is measured, while the underlying task and reference path remain identical across all methods.

Because the metric is based on dense logged samples rather than resampling every executed trajectory onto a common normalized arc-length grid, differences in sampling density may have a small influence on the numerical value of the mean error. However, since the same evaluation protocol was applied uniformly to all methods and the logged trajectories are dense in all cases, the reported comparison remains informative for relative performance assessment.

63 $${\mathrm{E}}_{\mathrm{i}}=\sqrt{{\left({x}_{s}- {x}_{c}\right)}^{2}+ {\left({y}_{s}- {y}_{c}\right)}^{2}+ {\left({z}_{s}- {z}_{c}\right)}^{2}}$$

64 $$\mathrm{M}\mathrm{e}\mathrm{a}\mathrm{n} \mathrm{E}\mathrm{r}\mathrm{r}\mathrm{o}\mathrm{r}=\frac{1}{n}\sum_{i=1}^{\mathrm{n}}{\mathrm{E}}_{i}$$

where ${\mathrm{E}}_{\mathrm{i}}$ is the error per sample, (${\mathrm{x}}_{\mathrm{s}}$, ${\mathrm{y}}_{\mathrm{s}}$, ${\mathrm{z}}_{\mathrm{s}}$) the set-point position, (${\mathrm{x}}_{\mathrm{c}}$, ${\mathrm{y}}_{\mathrm{c}}$, ${\mathrm{z}}_{\mathrm{c}}$) the current position, and $n$ denotes the number of logged actual end-effector samples recorded during one trajectory execution, not the number of nominal reference set-points. The reference trajectory itself always contains 51 set-points for every method.

*B. Repeated-run protocol*

To support reproducibility, each algorithm was executed for 20 independent runs for each reference trajectory (2D triangle and 3D helix). For stochastic methods (ACO, ACO–SQP, and ACO–B&B–SQP), runs used different random seeds; deterministic methods (SQP, B&B, and B&B–SQP) were repeated under identical settings to capture numerical/solver variability. For each method, we report the mean over 20 runs for (i) trajectory execution time and (ii) mean path error. In addition, within each run we compute the standard deviation of the instantaneous tracking error ${\mathrm{E}\mathrm{r}\mathrm{r}\mathrm{o}\mathrm{r}}_{i}$ over the $n$ logged samples to describe error dispersion along the trajectory; this within-run standard deviation is distinct from run-to-run variability.

*C. Statistical treatment*

The percentage changes reported below are descriptive mean differences. To avoid overstating performance, run-level mean path errors from the 20 repeated runs were considered as independent observations, and pairwise comparisons against SQP were estimated using Welch’s t-test with unequal variances, together with 95% confidence intervals and Hedges’ $g$ effect sizes. Accordingly, the percentage reductions reported in this section are interpreted as descriptive summaries of average performance and not, by themselves, as statements of statistical significance.

*D. Statistical definitions and mathematical rules used in the results section*

In this section, the reported error and statistical quantities are defined as follows. Let $Erro{r}_{i}$ denote the instantaneous geometric tracking error at sample $i$, defined by (63). For one trajectory execution containing $n$ logged actual path samples, the run-level mean path error is given by (64).

**Within-run standard deviation:** The quantity Std. Dev. (mm) reported in Table 7 denotes the standard deviation of the instantaneous tracking error within a single run, computed over the $n$ logged samples as

65 $${s}_{within}=\sqrt{\frac{1}{n-1}\sum_{i=1}^{n}({E}_{i}-Mean\hspace{0.25em}Error{)}^{2}}$$

This quantity describes the dispersion of the pointwise tracking error along one trajectory execution.

**Run-to-run standard deviation across 20 runs:** Suppose one method is repeated over $R=20$ independent runs, and let ${\stackrel{\_}{E}}_{r}$ denote the run-level mean path error of run $r$, where $r=1,2,\dots ,R$. The average mean path error across 20 runs is

66 $$\stackrel{\_}{x}=\frac{1}{R}\sum_{r=1}^{R}{\stackrel{\_}{E}}_{r}$$

The quantity **Std. Dev. across 20 runs (mm)** reported in Table 7 denotes the run-to-run standard deviation of the run-level mean path error, computed as

67 $${s}_{runs}=\sqrt{\frac{1}{R-1}\sum_{r=1}^{R}({\stackrel{\_}{E}}_{r}-\stackrel{\_}{x}{)}^{2}}$$

This quantity describes the variability in average performance between repeated runs of the same method.**Mean difference relative to SQP:** For pairwise comparison against SQP, the mean difference in average path error is defined as

68 $$\Delta ={\stackrel{\_}{x}}_{method}-{\stackrel{\_}{x}}_{SQP}$$

where $\overline{x}_{{{\mathrm{method}}}}$ is the mean path error of the compared method over 20 runs and $\overline{x}_{{{\mathrm{SQP}}}}$ is the corresponding mean path error of SQP over 20 runs. A negative value of $\Delta$ indicates that the compared method achieved lower mean path error than SQP.

**Standard error for Welch’s comparison:** Let ${\mathrm{s}}_{method}$ and ${\mathrm{s}}_{SQP}$ denote the run-to-run standard deviations of the mean path error for the compared method and SQP, respectively, and let ${\mathrm{n}}_{method}={\mathrm{n}}_{SQP}=20$. The standard error of the mean difference is

69 $$SE=\sqrt{\frac{{s}_{method}^{2}}{{n}_{method}}+\frac{{s}_{SQP}^{2}}{{n}_{SQP}}}$$

**Welch’s t-statistic:** The test statistic for pairwise comparison against SQP is

70 $$t=\frac{\Delta }{SE}$$

with Welch-Satterthwaite degrees of freedom

71 $$\nu =\frac{{\left(\frac{{s}_{method}^{2}}{{n}_{method}}+\frac{{s}_{SQP}^{2}}{{n}_{SQP}}\right)}^{2}}{\frac{{\left(\frac{{s}_{method}^{2}}{{n}_{method}}\right)}^{2}}{{n}_{method}-1}+\frac{{\left(\frac{{s}_{SQP}^{2}}{{n}_{SQP}}\right)}^{2}}{{n}_{SQP}-1}}$$

**95% confidence interval:** The 95% confidence interval for the mean difference is computed as

72 $$\Delta \pm {t}_{0.975,\nu }SE$$

where, ${t}_{0.975,\nu }$ is the critical value of the $t$-distribution with $\nu$ degrees of freedom.

***p*-value:** The two-sided p-value is computed from the $t$-distribution as

73 $$p=2\left[1-{F}_{t}(\mid t\mid ;\nu )\right]$$

where, ${F}_{t}(\cdot ;\nu )$ is the cumulative distribution function of the $t$-distribution with $\nu$ degrees of freedom. In this work, ${\boldsymbol{p}}$-value are reported only as approximate inferential context because they were estimated from run-level summary statistics rather than raw run-level observations. Accordingly, *p* < 0.05 is not interpreted as definitive confirmatory evidence, but only as an indicator of the strength and direction of the observed difference under the present approximation.

**Hedges’ **${\boldsymbol{g}}$** effect size:** To quantify the magnitude of the difference in standardized form, Hedges’ $g$ is used. First, the pooled standard deviation is computed as

74 $$s_{p} = \sqrt {\frac{{\left( {n_{{{\mathrm{method}}}} - 1} \right)s_{{{\mathrm{method}}}}^{2} + \left( {n_{SQP} - 1} \right)s_{{{\mathrm{SQP}}}}^{2} }}{{n_{{{\mathrm{method}}}} + n_{{{\mathrm{SQP}}}} - 2}}}$$

Then Cohen’s $d$ is

75 $$d = \frac{{\overline{x}_{{{\mathrm{method}}}} - \overline{x}_{{{\mathrm{SQP}}}} }}{{s_{p} }}$$

and Hedges’ correction factor is

76 $$J=1-\frac{3}{4({n}_{method}+{n}_{SQP})-9}$$

Thus, Hedges’ $g$ is

77 $$g=Jd$$

The sign of $g$ follows the sign of $\Delta$. A negative value indicates lower mean path error than SQP. As a practical guide, $\mid g\mid \approx 0.2$, $\mid g\mid \approx 0.5$, and $\mid g\mid \approx 0.8$ are commonly interpreted as small, medium, and large effects, respectively.

The inferential statistics reported here were estimated from the run-level summary statistics in Table 7, assuming that the reported ‘Std. Dev. across 20 runs’ corresponds to the run-to-run standard deviation of the mean path error over 20 independent runs per method. Therefore, the reported confidence intervals, *p*-values, and effect sizes should be interpreted as approximate inferential estimates rather than definitive confirmatory results, unless verified using the raw run-level observations.

Based on this repeated-run analysis, the percentage reductions in mean path error reported in this section should be interpreted as descriptive differences in sample means rather than, by themselves, as statements of statistically confirmed superiority. The inferential statistics reported here were estimated from run-level summary statistics rather than analyzed directly from the raw run-level observations; therefore, they should be interpreted as approximate. Under this interpretation, the observed reductions in mean path error are best treated as descriptive trends within the present experimental setup, and broader claims of statistically repeatable superiority should be avoided unless confirmed from the raw run-level data.

*E. Descriptive results from *Table 7

Although $n$ varies across methods, the comparison remains fair because all methods are evaluated on the same nominal reference trajectory with the same 51 set-points, under the same robot platform and the same error-computation procedure; the variation in $n$ reflects only how densely the realized path was logged during execution.

To improve reproducibility, the final hyperparameter settings and their local sensitivity trends are summarized in Table 6. The same settings were used for all runs and both trajectories, so the reported comparisons reflect algorithm-family behavior under a fixed common configuration rather than case-specific retuning.

The two path shapes on which the methods were evaluated include the 3D helix and the 2D triangle. **For the triangle trajectory**, B&B showed a slightly lower average mean path error than SQP, with a descriptive difference of 1.86%, while requiring 1.88% more execution time. Among the standalone methods, ACO showed the lowest average mean path error, with a descriptive reduction of 17.90% relative to SQP, at the cost of 22.08% higher execution time. The hybrid B&B–SQP method showed a descriptive reduction in average mean path error of 3.87% with a 4.15% increase in execution time, while ACO–SQP showed a descriptive reduction of 11.58% with a 13.08% increase in time. Under the present experimental setup, the proposed ACO–B&B–SQP method achieved the lowest average mean path error for the 2D triangle trajectory, decreasing from 2.53194 mm for SQP to 1.84441 mm, corresponding to a descriptive difference of 27.15%. Relative to standalone ACO, the proposed method also showed a descriptively lower mean path error by 11.28% and a lower execution time by 12.44%. These differences are reported descriptively. The repeated-run analysis provides additional statistical context for the observed mean-error differences relative to SQP, but in the present manuscript these differences are interpreted conservatively as descriptive trends within the evaluated setup.

**For the 3D helix trajectory**, B&B again showed a slightly lower average mean path error than SQP, with a descriptive difference of 3.16%, while requiring 2.23% more execution time. ACO showed a descriptive reduction in average mean path error of 14.86% relative to SQP, with execution time higher by 16.84%. The B&B–SQP hybrid showed a descriptive reduction of 3.32% in average mean path error with a 3.18% increase in execution time, while ACO–SQP showed a descriptive reduction of 9.07% with a 9.87% increase in time. For this trajectory as well, the proposed ACO–B&B–SQP method achieved the lowest average mean path error under the present setup, decreasing from 2.40781 mm for SQP to 1.87155 mm, corresponding to a descriptive difference of 22.27%. Relative to standalone ACO, it showed a descriptively lower mean path error by 8.71% together with a 9.39% lower execution time. As with the triangle trajectory, the repeated-run analysis provides additional statistical context for the observed mean-error differences relative to SQP; however, in the present manuscript these differences are interpreted conservatively as descriptive trends within the evaluated setup.

*F. Pairwise statistical comparison against SQP*

**For the 2D triangle trajectory**, all methods produced lower average mean path error than SQP, with the largest descriptive reduction observed for the proposed ACO-B&B-SQP method. Specifically, B&B showed $\Delta =-0.047$ mm, 95% CI [− 0.209, 0.115], $p=0.558$, and $g=-0.183$; ACO showed $\Delta =-0.453$ mm, 95% CI [− 0.608, − 0.298], $p=7.58{\times{x}}{10}^{-7}$, and $g=-1.836$; ACO-SQP showed $\Delta =-0.293$ mm, 95% CI [− 0.452, − 0.135], $p=5.9{\times{x}}{10}^{-4}$, and $g=-1.163$; B&B-SQP showed $\Delta =-0.098$ mm, 95% CI [− 0.258, 0.062], $p=0.224$, and $g=-0.383$; and the proposed ACO-B&B-SQP showed $\Delta =-0.688$ mm, 95% CI [− 0.839, − 0.536], $p=4.48{\times{x}}{10}^{-11}$, and $g=-2.846$. These numerical comparisons suggest lower average mean path error for several methods relative to SQP under the present setup. However, because the inferential quantities are estimated from summary statistics rather than directly from the raw run-level observations, the present manuscript interprets these differences conservatively as descriptive trends rather than as standalone proof of statistically established superiority.

**For the 3D helix trajectory**, several methods again showed lower average mean path error than SQP, with the largest descriptive reduction observed for the proposed ACO-B&B-SQP method. Specifically, B&B showed $\Delta =-0.076$ mm, 95% CI [− 0.216, 0.064], $p=0.279$, and $g=-0.341$; ACO showed $\Delta =-0.358$ mm, 95% CI [− 0.495, − 0.220], $p=5.73{\times{x}}{10}^{-6}$, and $g=-1.634$; ACO-SQP showed $\Delta =-0.218$ mm, 95% CI [− 0.357, − 0.079], $p=0.00292$, and $g=-0.986$; B&B-SQP showed $\Delta =-0.080$ mm, 95% CI [− 0.221, 0.062], $p=0.260$, and $g=-0.354$; and the proposed ACO-B&B-SQP showed $\Delta =-0.536$ mm, 95% CI [− 0.672, − 0.401], $p=1.2{\times{x}}{10}^{-9}$, and $g=-2.482$. As in the triangle case, these comparisons numerically favor the ACO-based methods under the present setup. Nevertheless, the inferential quantities should be viewed as approximate because they were estimated from run-level summary statistics, and the manuscript therefore interprets the observed differences conservatively as descriptive performance trends.

Across both trajectories, the proposed ACO-B&B-SQP method yielded the lowest average mean path error among the tested methods under the present setup. The pairwise comparisons provide useful statistical context for the direction and approximate magnitude of the observed differences relative to SQP. However, because these inferential quantities were estimated from run-level summary statistics rather than directly from the raw run-level observations, the present study avoids treating them as the sole basis for strong confirmatory claims. Accordingly, terms such as “reduction” and “improvement” are interpreted in this manuscript as descriptive differences in average performance under the evaluated conditions.

*G. Interpretation of method behavior*

**Why ACO may show lower average path error:** The repeated observation that ACO shows one of the lower average geometric errors among the individual methods may be related to its global search over discrete IK/branch and set-point assignment choices. In redundant or multi-solution IK, different branches can have very different conditioning and tracking behavior; by exploring a larger combinatorial space, ACO is more likely to find sequences that avoid near-singularities, reduce joint discontinuities between set points, and produce smoother motion, each lowering geometric deviation.

One possible explanation is that ACO is not required to identify the exact optimum on the discretized grid. In the present framework, it only needs to identify promising seed regions or favorable IK branches, after which continuous local refinement can recover additional accuracy. This helps explain why a moderate discretization may still be practically effective despite the large nominal combinatorial space.

**Why SQP is faster but can be less accurate:** By comparison, SQP is a local continuous method and can be initialization-sensitive in nonconvex problems. If started near a suboptimal IK branch or timing allocation, it may converge quickly to a locally optimal but less accurate solution. This plausibly explains why SQP is consistently fastest yet shows higher mean error in Table 7. A compact supporting test is to run SQP from multiple IK seeds and report the outcome spread.

**B&B reduced initialization sensitivity:** Because B&B searches over discrete choices, it is typically less sensitive to continuous initialization than SQP. Its performance, however, depends on the discrete formulation, discretization granularity, pruning bounds, and whether smoothness costs are included. Coarser discretization or aggressive pruning can reduce runtime but may exclude higher-quality sequences. Thus, B&B’s advantage should be interpreted primarily as reduced initialization sensitivity (relative to SQP), and Table 8 indicates that the B&B- and B&B-SQP-based comparisons do not show clear evidence of lower mean path error relative to SQP under the present repeated-run analysis.

**Table 8:** Pairwise statistical comparison of mean path error against SQP.

shape	Method	Δ (mm)	95% CI (mm)	$p$-value	Hedges’ ($g$)
2D Triangle	B&B	− 0.047	[− 0.209, 0.115]	0.558	− 0.183
	ACO	− 0.453	[− 0.608, − 0.298]	$7.58 \times 10^{ - 7}$	− 1.836
	ACO-SQP	− 0.293	[− 0.452, − 0.135]	$5.9 \times 10^{ - 4}$	− 1.163
	B&B-SQP	− 0.098	[− 0.258, 0.062]	0.224	− 0.383
	ACO-B&B-SQP	− 0.688	[− 0.839, − 0.536]	$4.48 \times 10^{ - 11}$	− 2.846
3D Helix	B&B	− 0.076	[− 0.216, 0.064]	0.279	− 0.341
	ACO	− 0.358	[− 0.495, − 0.220]	$5.73 \times 10^{ - 6}$	− 1.634
	ACO-SQP	− 0.218	[− 0.357, − 0.079]	0.00292	− 0.986
	B&B-SQP	− 0.080	[− 0.221, 0.062]	0.2	− 0.354
	ACO-B&B-SQP	− 0.536	[− 0.672, − 0.401]	$1.2 \times 10^{ - 9}$	− 2.482

In Table 8, $\Delta$ is computed by (68) and denotes the difference in average mean path error relative to SQP. The 95% CI is computed by (72), $p$ is computed by (73), and $g$ is computed by (77). Negative $\Delta$ values indicate lower mean path error than SQP. Because these inferential quantities were reconstructed from run-level summary statistics rather than computed directly from raw run-level observations, they are reported only as approximate statistical context and should not be interpreted as standalone confirmatory evidence.

**The hybrids follow naturally:** the discrete stage (ACO or B&B) selects a good IK/sequence configuration, then SQP refines continuous variables (e.g., timing and smoothness) efficiently. In the proposed Hybrid ACO-B&B-SQP framework, the complementary strengths of ACO, B&B, and SQP are integrated in a single optimization pipeline: ACO enhances global exploration, B&B improves discrete decision quality and search reliability, and SQP performs efficient local refinement. This combined strategy may help explain why the proposed method consistently showed the lowest average geometric path error among the tested methods, and the present repeated-run analysis is numerically consistent with the observation that ACO-B&B-SQP achieved the lowest average mean path error under the evaluated conditions. In this manuscript, however, that result is interpreted conservatively as a descriptive outcome of the present comparative setup rather than as a universal statistical guarantee.

*H. Practical significance*

Although the absolute differences in mean path error are relatively small (approximately 0.54–0.69 mm when comparing the proposed Hybrid ACO-B&B-SQP method with SQP), they correspond to relative improvements of up to 27.15% for the 2D triangle trajectory and 22.27% for the 3D helix trajectory compared with SQP. The practical significance of these improvements depends on the specific application tolerance (e.g., precision assembly versus painting or polishing). Therefore, the results should be interpreted in relation to the required task tolerance. In particular, since the absolute improvement is on the order of a few tenths of a millimeter, its practical relevance should be evaluated against the system’s overall error budget, including calibration residuals, sensing and estimation noise, and controller tracking limitations, as well as the allowable geometric deviation of the task. Notably, the proposed Hybrid ACO-B&B-SQP method produced the lowest average mean path error among the tested methods on both trajectories under the present setup. In applications where sub-millimeter differences are operationally relevant, even these observed reductions may be practically meaningful, but that practical meaning should be judged relative to task tolerance and overall system uncertainty.

**Overall**, Table 7 suggests an accuracy–speed trade-off across the tested methods. SQP (or B&B–SQP) is more suitable when cycle time is the primary concern, whereas ACO (or ACO–SQP) is preferable when higher tracking accuracy is prioritized. In continuous optimization, SQP maintains a good balance between computational efficiency and solution accuracy. Among all tested methods, the proposed Hybrid ACO-B&B-SQP showed the lowest average mean path error under the present setup, while its runtime remained higher than SQP but lower than standalone ACO. Consistent with Table 7, the ACO-based methods show the largest observed reductions in average mean path error relative to SQP under the present setup, whereas the B&B-based methods show smaller observed differences. In this manuscript, these results are discussed primarily as descriptive performance trends under a fixed common evaluation framework.

*I. Run-to-run repeatability visualization*

**Fig. 20:**
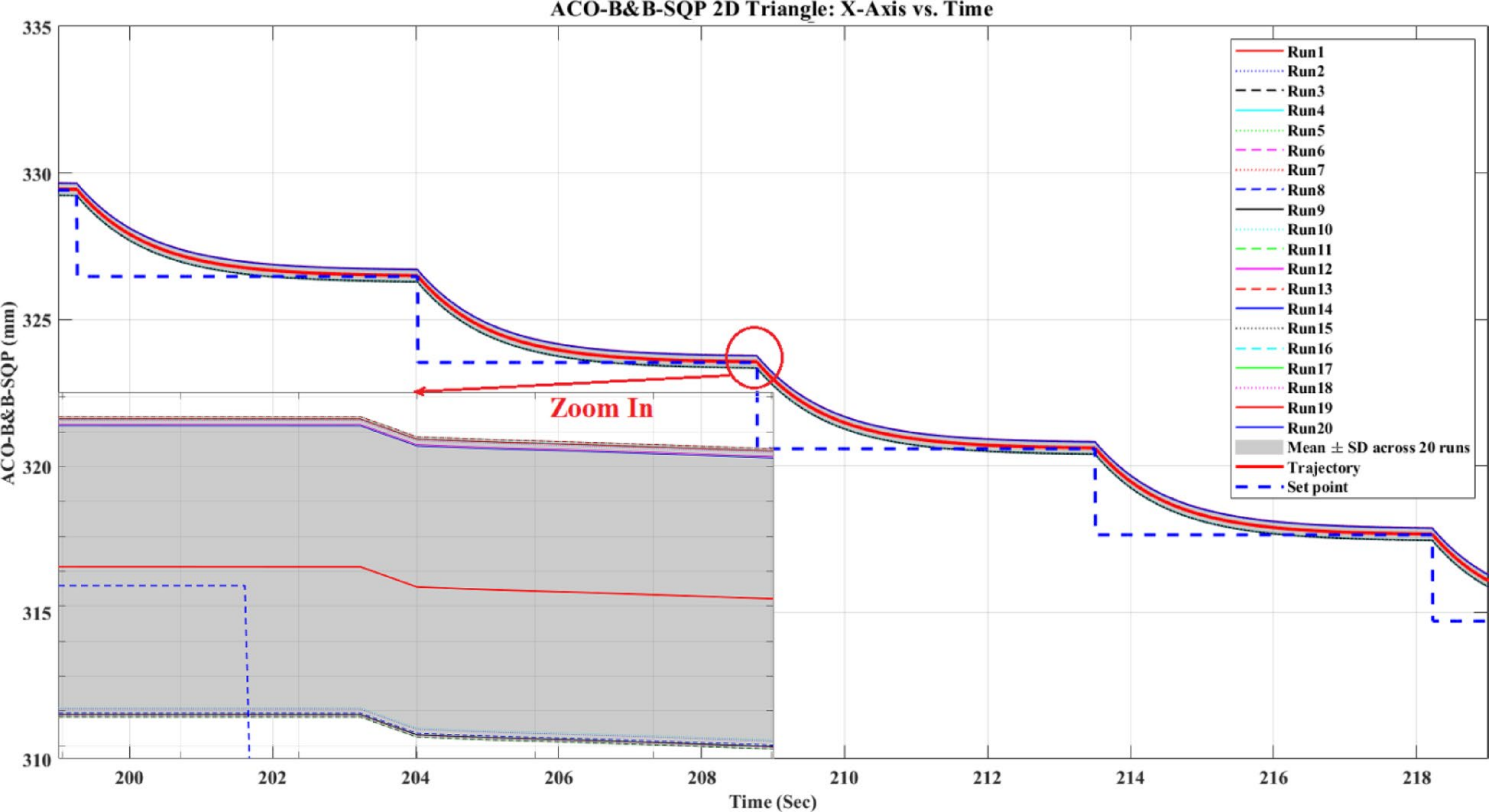
Zoomed 2D triangle X-axis repeatability, ACO-B&B-SQP, 20 runs.

**Fig. 21:**
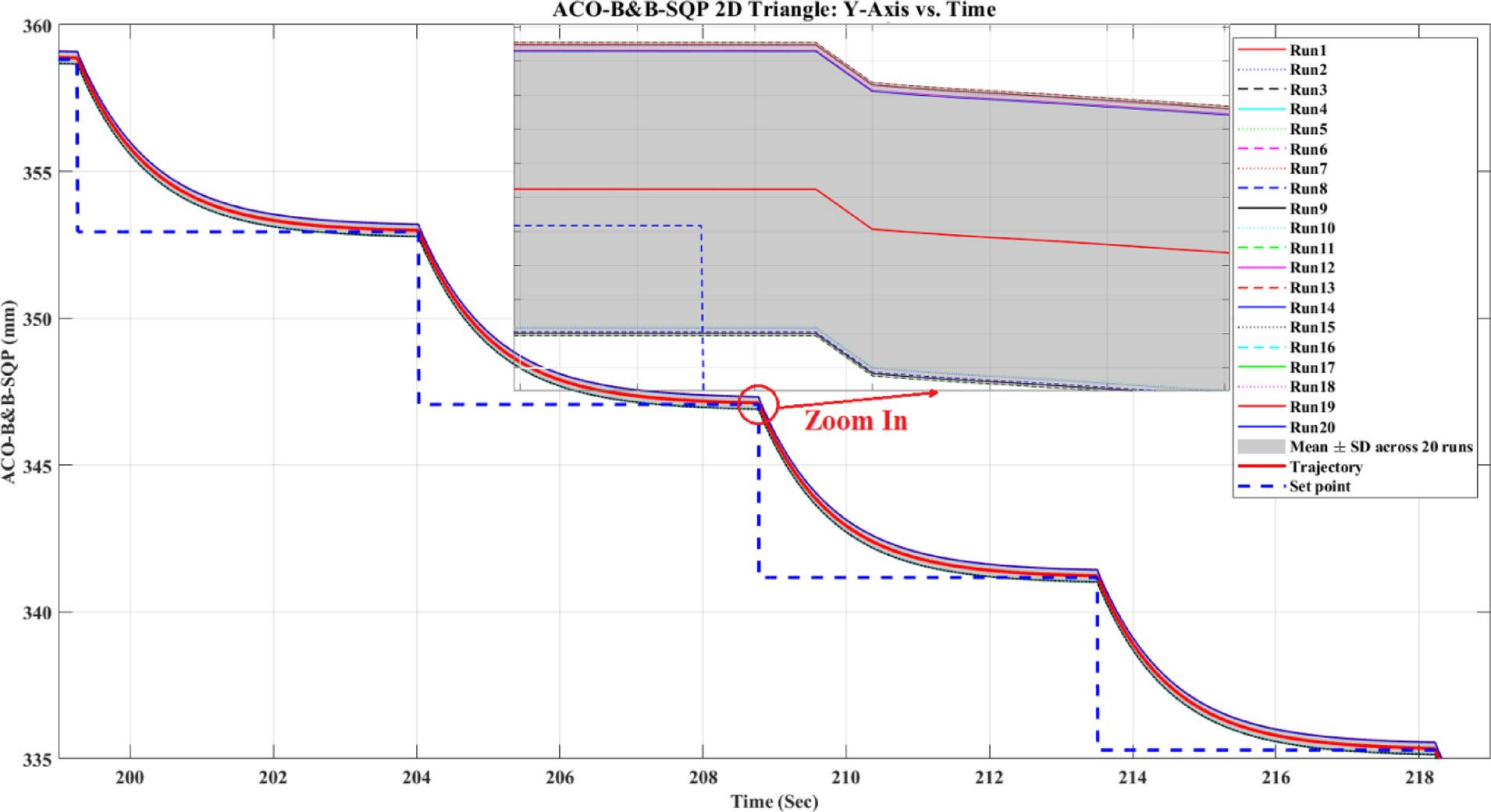
Zoomed 2D triangle Y-axis repeatability, ACO-B&B-SQP, 20 runs.

**Fig. 22:**
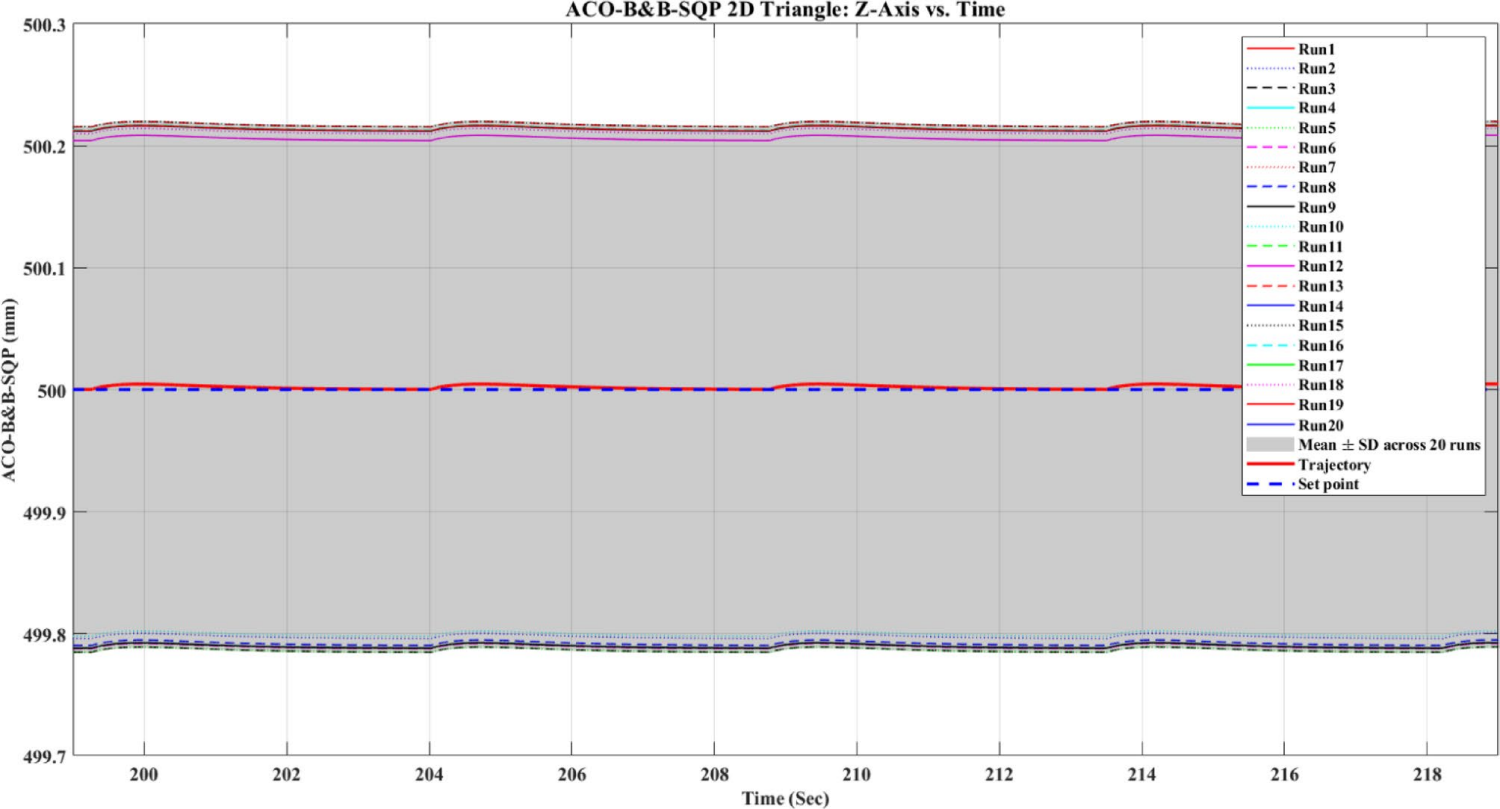
Zoomed 2D triangle Z-axis repeatability, ACO-B&B-SQP, 20 runs.

**Fig. 23:**
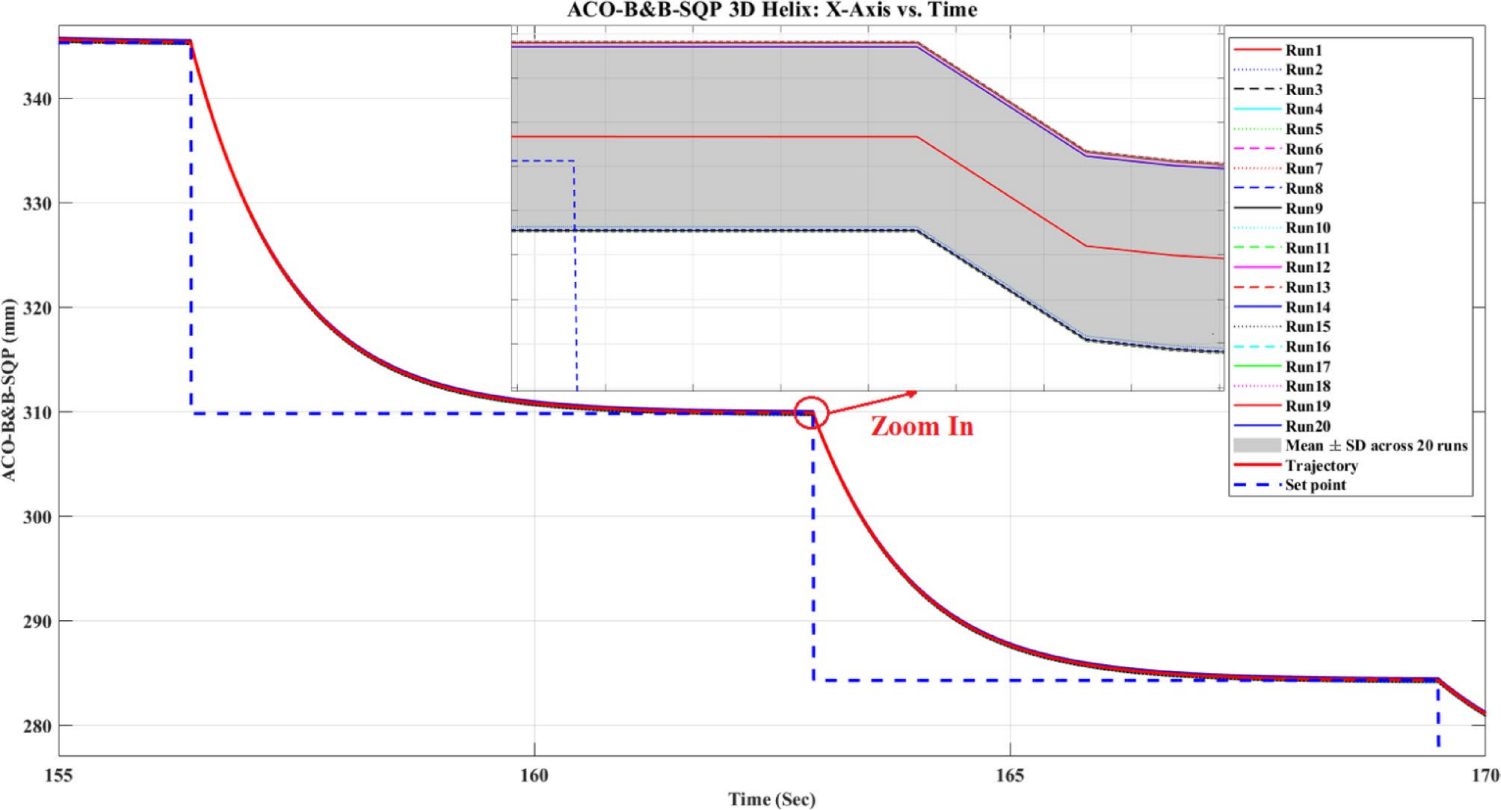
Zoomed 3D helix X-axis repeatability, ACO-B&B-SQP, 20 runs.

**Fig. 24:**
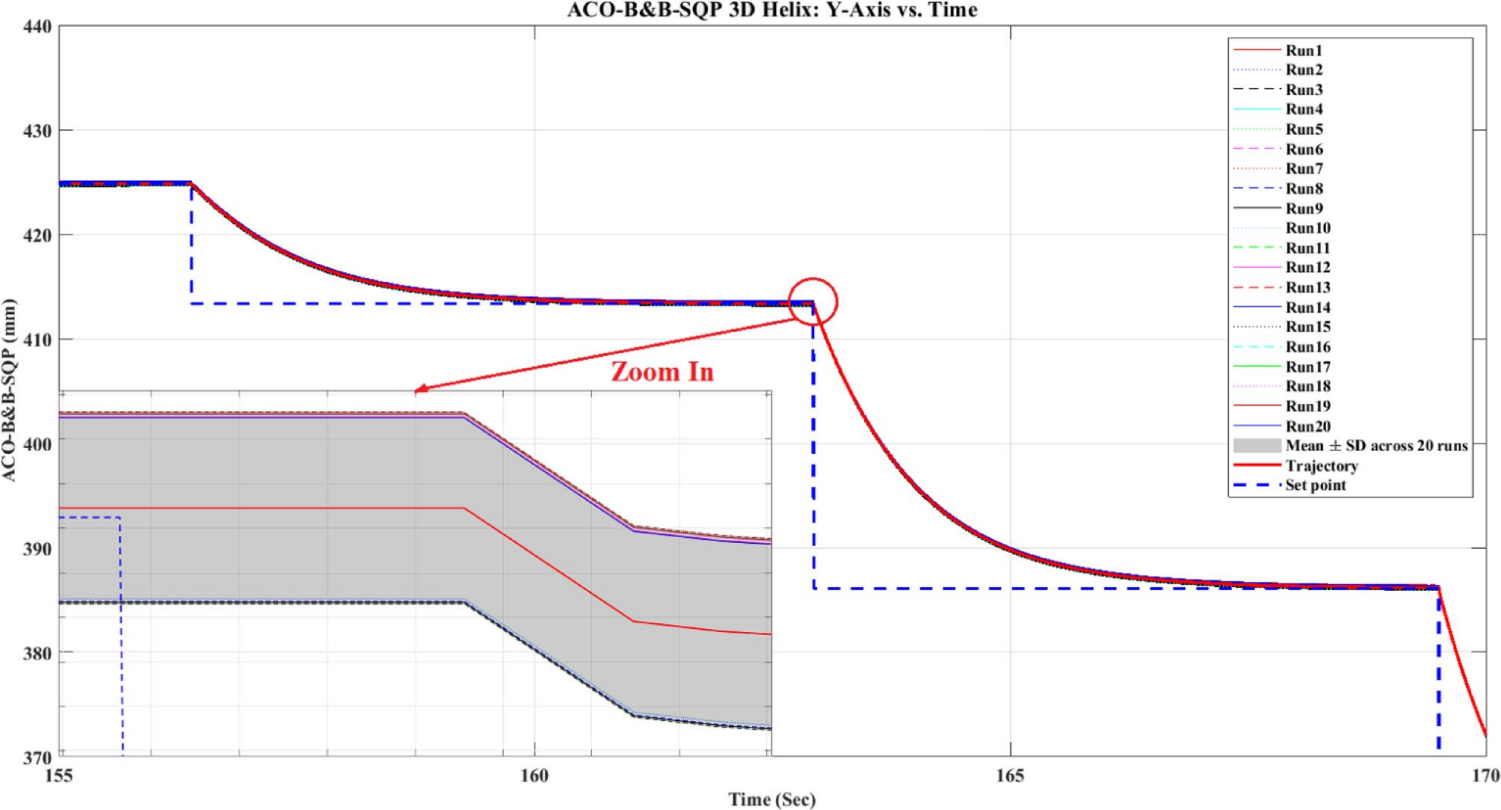
Zoomed 3D helix Y-axis repeatability, ACO-B&B-SQP, 20 runs.

**Fig. 25:**
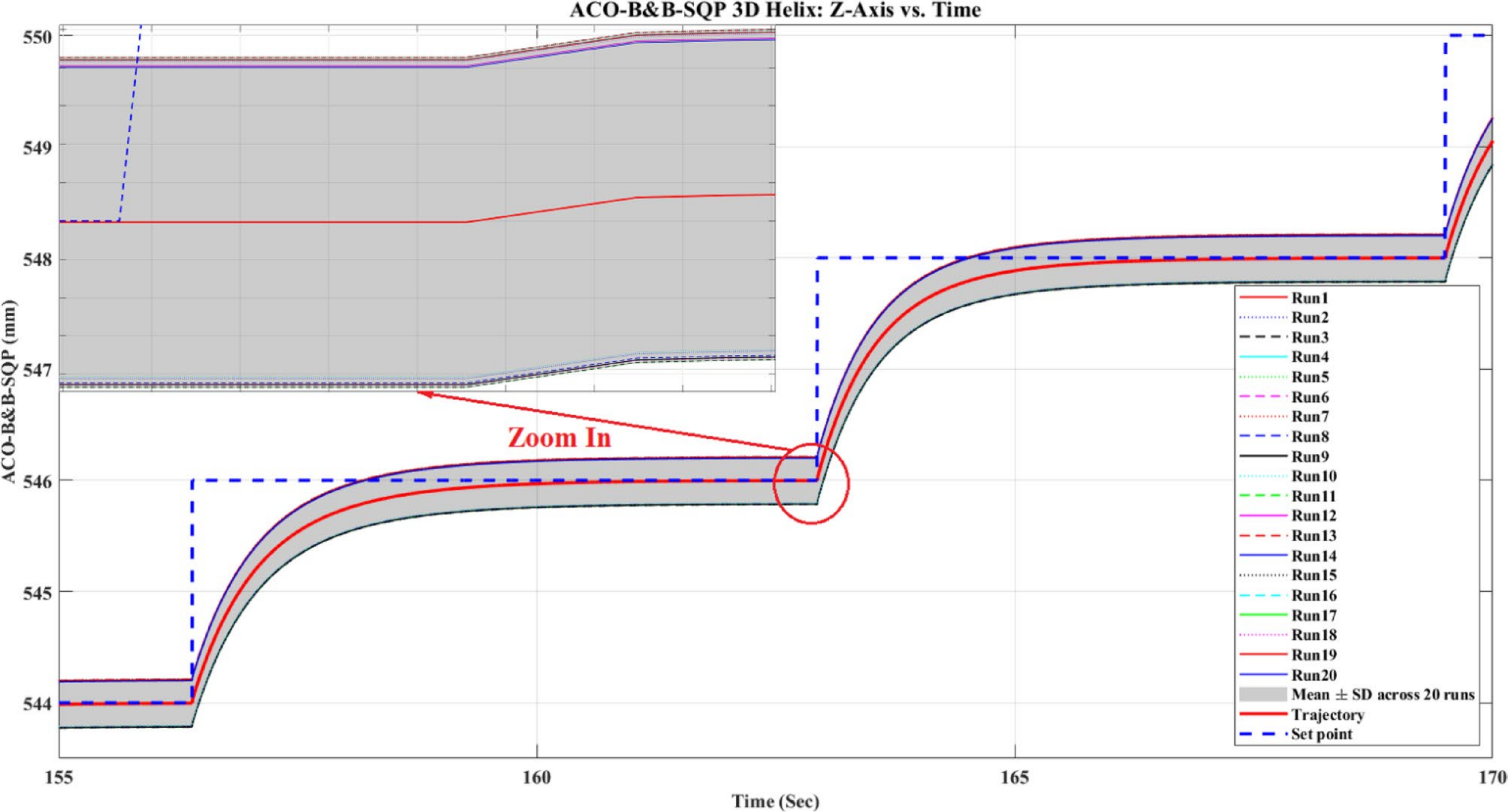
Zoomed 3D helix Z-axis repeatability, ACO-B&B-SQP, 20 runs.

To complement the axis-wise tracking curves in Figs. 14, 15, 16, 17, 18, 19, 20, 21, 22, 23, 24 and 25 provide a visual repeatability presentation for the proposed ACO-B&B-SQP method over 20 repeated runs for the X, Y, and Z coordinates of the 2D triangle and 3D helix trajectories. In these figures, the thin curves correspond to the individual runs, while the shaded region provides a visual indication of variability around the mean trajectory. Because the full-scale axis plots cause substantial overlap of the repeated curves and make the spread difficult to observe, zoomed views are included around the most informative transition regions. To preserve readability, repeatability plots with variability bands are shown separately only for the best-performing method, ACO-B&B-SQP, whereas the numerical run-to-run variability for all compared methods is reported in Table 7 Results Analysis through the “Std. Dev. across 20 runs” of the run-level mean path error. The added plots show that the run-to-run spread remains narrow across all axes, with the most visible variation occurring mainly near trajectory transitions, whereas the steady segments exhibit near-complete overlap of the repeated runs.

## Conclusion and future work

The comparative results provide guidance on the runtime-accuracy trade-off among the evaluated IK methods under the present setup. This study used six different techniques to optimize the 6-DOF Viper 650 s robot’s inverse kinematics for surgical placement and suturing: standalone Sequential Quadratic Programming (SQP), Branch and Bound (B&B), Ant Colony Optimization (ACO), hybrid ACO with SQP, hybrid B&B with SQP, and the proposed hierarchical three-stage hybrid ACO–B&B–SQP, within a unified evaluation setup. The main contribution of the study is therefore twofold: first, a controlled and unified comparative framework for evaluating six IK optimization methods under identical modeling assumptions, constraints, trajectories, and evaluation metrics; second, the implementation and evaluation of the ACO-B&B-SQP three-stage pipeline within the same framework. Two trajectories, a 2D triangle and a 3D helix, were used to evaluate the effectiveness of the six IK solutions.

Under the present experimental conditions, SQP achieved the fastest runtime (approximately 226 s for the 2D triangle), whereas the proposed Hybrid ACO-B&B-SQP achieved the lowest mean path error overall. Among the standalone methods, ACO yielded the lowest mean geometric path error, while SQP remained the fastest. Across all evaluated methods, including the hybrids, the proposed Hybrid ACO-B&B-SQP consistently produced the lowest mean path error on both trajectories. B&B also appeared less sensitive to continuous initialization than SQP because it explores multiple regions of the bounded joint space. Relative to SQP, the proposed Hybrid ACO-B&B-SQP showed descriptive reductions in average mean path error of 27.15% for the 2D triangle and 22.27% for the 3D helix under the present setup. The pairwise repeated-run analysis in Table 8 provides additional statistical context for these observed differences. However, because the inferential quantities were estimated from run-level summary statistics rather than directly from the raw run-level observations, the present work interprets these reductions conservatively as descriptive comparative outcomes within the evaluated conditions. Overall, the proposed Hybrid ACO-B&B-SQP is best interpreted as an accuracy-oriented candidate within the unified comparative framework developed in this work, whereas SQP remains preferable when cycle time is the dominant requirement.

Although a local sensitivity study was included for the principal ACO, B&B, and SQP parameters, a broader automated hyperparameter search across all methods remains outside the scope of the present work and could be explored in future extensions of this comparative study.

Future work should focus on improving the computational efficiency of the ACO-B&B-SQP pipeline, since the present results show that it provides the lowest mean path error but at a higher runtime than SQP. This can be pursued through adaptive discretization, more selective region refinement in the B&B stage, or continuous relaxation strategies before SQP refinement. In addition, the optimization framework should be extended to include application-specific surgical constraints, such as collision avoidance, restricted tool approach directions, minimum invasive angle requirements, and other safety margins within a multi-objective formulation. Another important direction is to apply the same comparative framework to redundant and higher-DOF manipulators, where multiple feasible IK branches are more prominent. Finally, future experiments should include representative classical baselines such as DLS and JPI, as well as widely used metaheuristics such as PSO and GA, under identical constraints and evaluation metrics to broaden comparative analysis.

## Data Availability

All data generated or analyzed during this study are included in this published article.
